# Supplementary material for: Impaired LXRα Phosphorylation Attenuates Progression of Fatty Liver Disease
Source: Cell Rep. 2019 Jan 22;26(4):984–995.e6. doi: 10.1016/j.celrep.2018.12.094 (PMC6344342; doi:10.1016/j.celrep.2018.12.094)
Supplement: Document S1. Figures S1–S6 and Tables S1–S4 [file mmc1.pdf]

Cell Reports, Volume *26*

Supplemental Information

Impaired LXRα Phosphorylation Attenuates Progression of Fatty Liver Disease

Natalia Becares, Matthew C. Gage, Maud Voisin, Elina Shrestha, Lucia Martin-Gutierrez, Ning Liang, Rikah Louie, Benoit Pourcet, Oscar M. Pello, Tu Vinh Luong, Saioa Goñi, Cesar Pichardo-Almarza, Hanne Røberg-Larsen, Vanessa Diaz-Zuccarini, Knut R. Steffensen, Alastair O'Brien, Michael J. Garabedian, Krista Rombouts, Eckardt Treuter, and Inés Pineda-Torra

Figure S1. Related to Figures 1 & 2

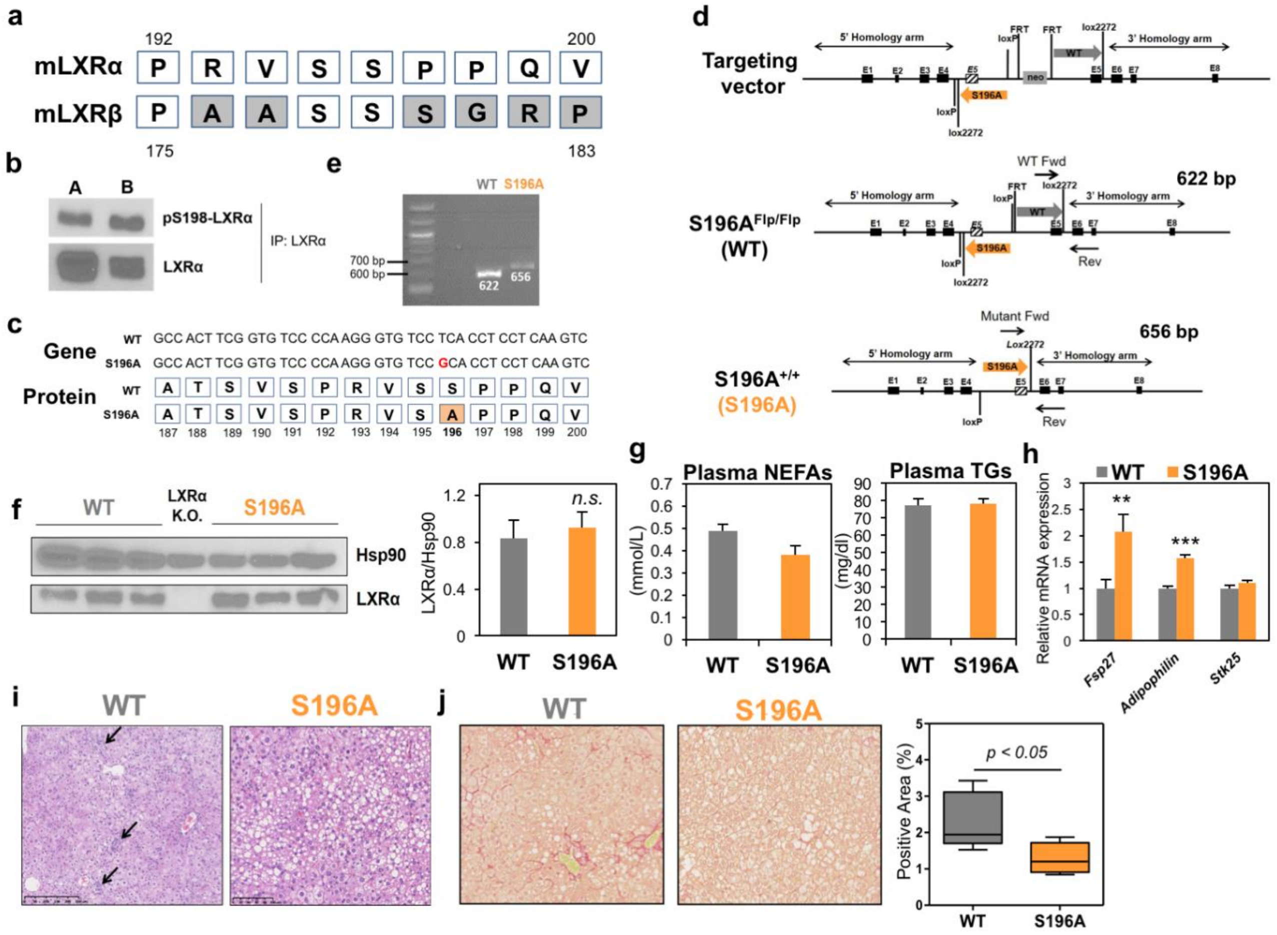

(a) Alignment of the murine LXRα and LXRβ showing differences in S196 phosphorylation motifs. **(b)** LXRα phosphorylation at Ser198 and total LXRα levels in human liver lysates (n=2) by immunoblotting. **(c)** WT and S196A genomic and protein sequence alignment of the murine LXRα depicting the single-site mutation at S196A. **(d)** Targeting construct containing the loxP and FRT sites, the predicted homologous recombinant alleles and the resulting WT and LXRα knock-in locus incorporating the mutated sequence. Diagram also shows oligos used for genotyping and product size. **(e)** Gel electrophoresis of DNA amplified products using the corresponding primers.

(f) Total LXRα and Hsp90 detected by immunoblotting in WT, S196A and LXRα knock-out livers. Densitometry quantification on LXRα levels normalised to Hsp90 loading control (n=3). (**g**) Plasma non-esterified fatty acids (NEFAs) and triglycerides (TGs) levels from WT and S196A mice on HFHC diet (n=5-6). Data are means ± SEM. **(h)** Hepatic gene expression of lipid droplet proteins from WT or S196A mice (n=6). Results shown normalized to cyclophilin and relative to WT set as 1. Data represents means ± SEM. * $p < 0.05$ or ** $p < 0.005$ relative to WT determined by Student's t-test. (**i**) Representative image of H&E-stained liver of WT and S196A mice fed a HFHC diet for 6 weeks. Arrows are pointing several inflammatory loci in WT liver. Scale bar at 50 μM. **(j)** Representative images of Picrosirius Red-stained liver sections from WT and S196A mice on a HFHC diet for 12 weeks. Images are at 200x magnifications. Quantification of Picrosirius red-stained areas on three independent areas per section (n=6). Data represent means ± SEM.

Figure S2. Related to Figures 2 & 3.

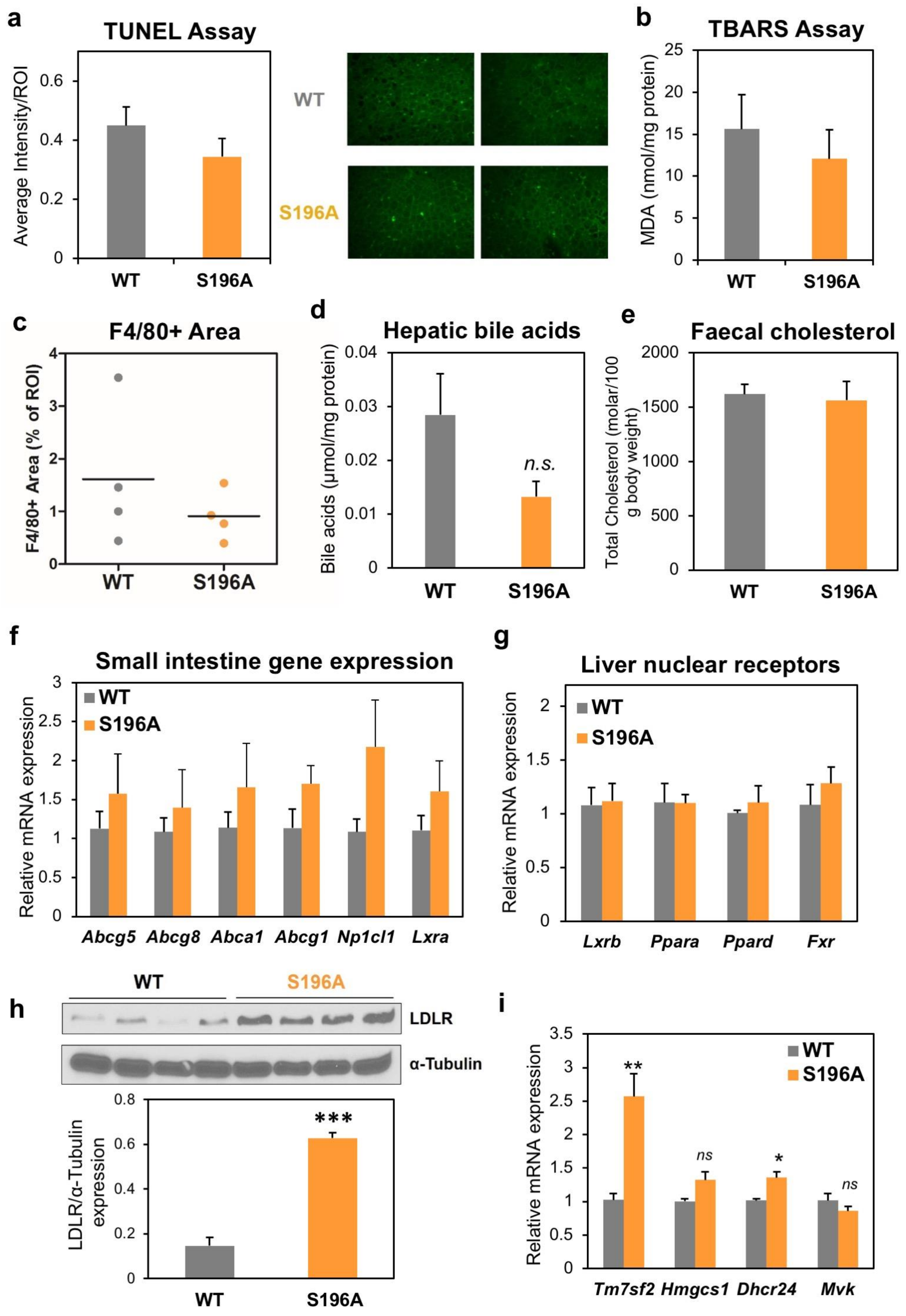

(a) Hepatic cell apoptosis assessed *in situ* by Direct DNA Fragmentation (TUNEL) Assay (n=6/group) *(Right)*. Representative images of TUNEL-stained liver sections from WT and S196A mice at 200x magnification (*Left).* **(b)** Hepatic lipid peroxidation shown as MDA levels in WT and S196A livers (n=6) normalised to protein levels in tissue homogenates. **(c)** Quantification of F4/80-positively stained areas in liver sections of WT and S196A mice (n=4) at 200x magnification. Dots represent average of three independent areas per animal. (**d**) Hepatic bile acid levels from WT and S196A mice fed a HFHC diet for 6 weeks (n=6). Values normalized to protein levels in liver homogenates. **(e)** Total cholesterol levels of faeces from WT and S196A mice (n=4). Values are shown per 100 g of dried faeces and normalized to animal body weight. **(f)** Small intestine and **(g)** **(i)** hepatic gene expression from WT or S196A mice fed a HFHC diet for 6 weeks (n=6). Results shown normalized to cyclophilin levels and relative to WT. **(h)** LDL-Receptor (LDLR) and α-Tubulin levels detected by immunoblotting in WT and S196A livers. Densitometry was performed on the levels of LDLR and normalised to the levels of the housekeeping α-Tubulin (n=4). Data represent means ± SEM. * $p < 0.05$ or ** $p < 0.005$ relative to WT determined by Student's t-test.

Figure S3. Related to Figure 4.

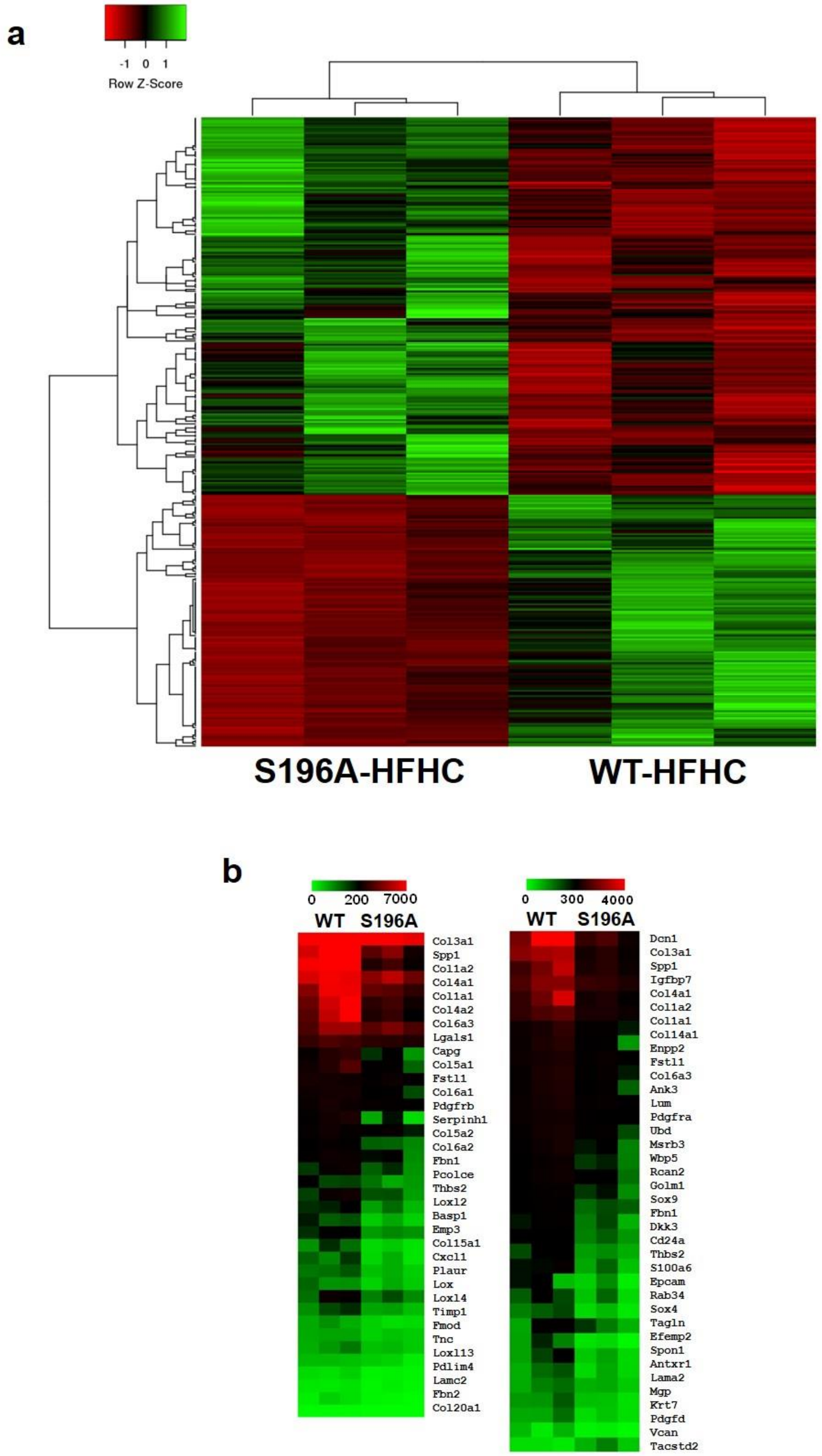

(a) Clustered heatmap of hepatic RNAseq normalised gene counts in WT and S196A mutant mice (n=3/genotype) of regulated genes in response to a HFHC diet. **(b)** Heatmaps of hepatic RNAseq normalised gene counts (n=3/genotype) for fibrosis (left) and mouse hepatic expression of genes previously identified to be part of a signature that distinguishes human NAFLD (right).

Figure S4. Related to Figure 4.

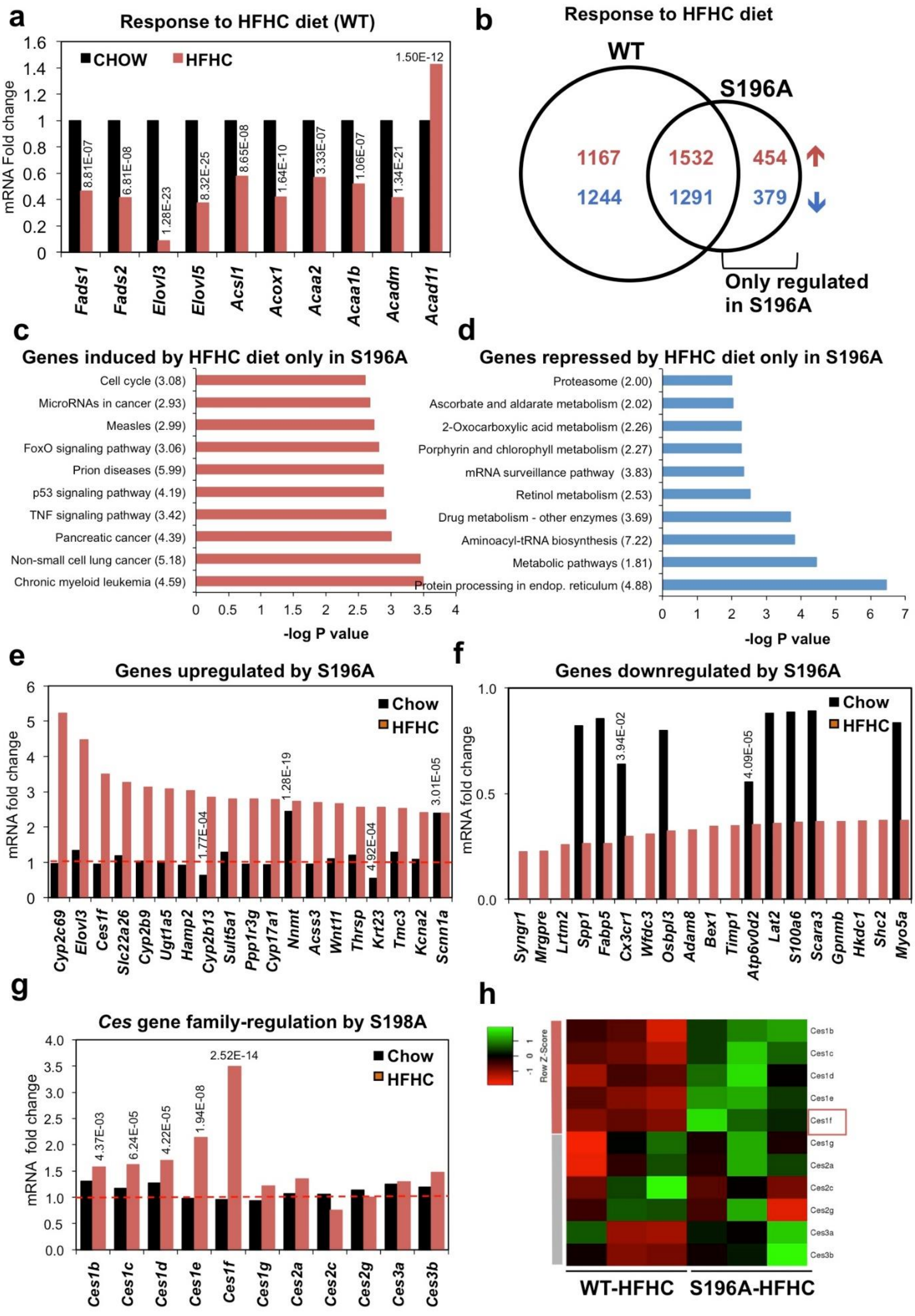

(a) Fold change of hepatic RNAseq gene counts of fatty acid genes in response to the HFHC diet compared to chow (set as 1) in WT mice (n=3/group). Adjusted *p* values (FDR 0.05) are shown. **(b)** Venn diagram of genes induced (red) or reduced (blue) in response to HFHC diet in WT or S196A mice. Significance set at $p \leq 0.05$. **(b,c)** Pathway analysis considering group of genes induced **(b)** or repressed **(c)** by HFHC diet only in S196A mice. Bar graphs shows top ten KEGG Pathways with enrichment score between brackets. **(d)** Fold change of hepatic RNAseq normalised gene counts of top upregulated genes in S196A compared to WT mice (from Fig. 4) on chow or HFHC diet (n=3/genotype). Shown are p values of genes differentially expressed between WT and S196A mice on chow. All genes shown are significantly regulated on the HFHC diet ($p<0.05$). Red bar set at fold change=1 indicates no change in gene expression between WT and S196A mice. **(e)** Fold change of hepatic RNAseq normalised gene counts of top downregulated genes in S196A compared to WT mice (from Fig. 4) fed chow or HFHC diet (n=3/genotype). For gene expression on chow, p values of genes differentially expressed between WT and S196A are shown. Data is not shown for those genes minimally expressed in chow. For gene expression on the HFHC diet, all genes depicted are significantly reduced ($p<0.05$). **(g)** Fold change of hepatic RNAseq normalised gene counts for Ces gene family members comparing WT and S1986A genotypes by diet (n=3/group). Shown are p values of genes differentially expressed on a HFHC diet. Red bar set at fold change=1 indicates no change in gene expression between WT and S196A mice. **(h)** Heatmap of hepatic RNAseq normalised gene counts in WT and S196A mutant mice (n=3/genotype) of Ces family genes on a HFHC diet. Red and grey bars indicate positively regulated or unchanged genes, respectively. Highest regulated Ces member (Ces1f) is shown boxed.

Figure S5. Related to Figure 5.

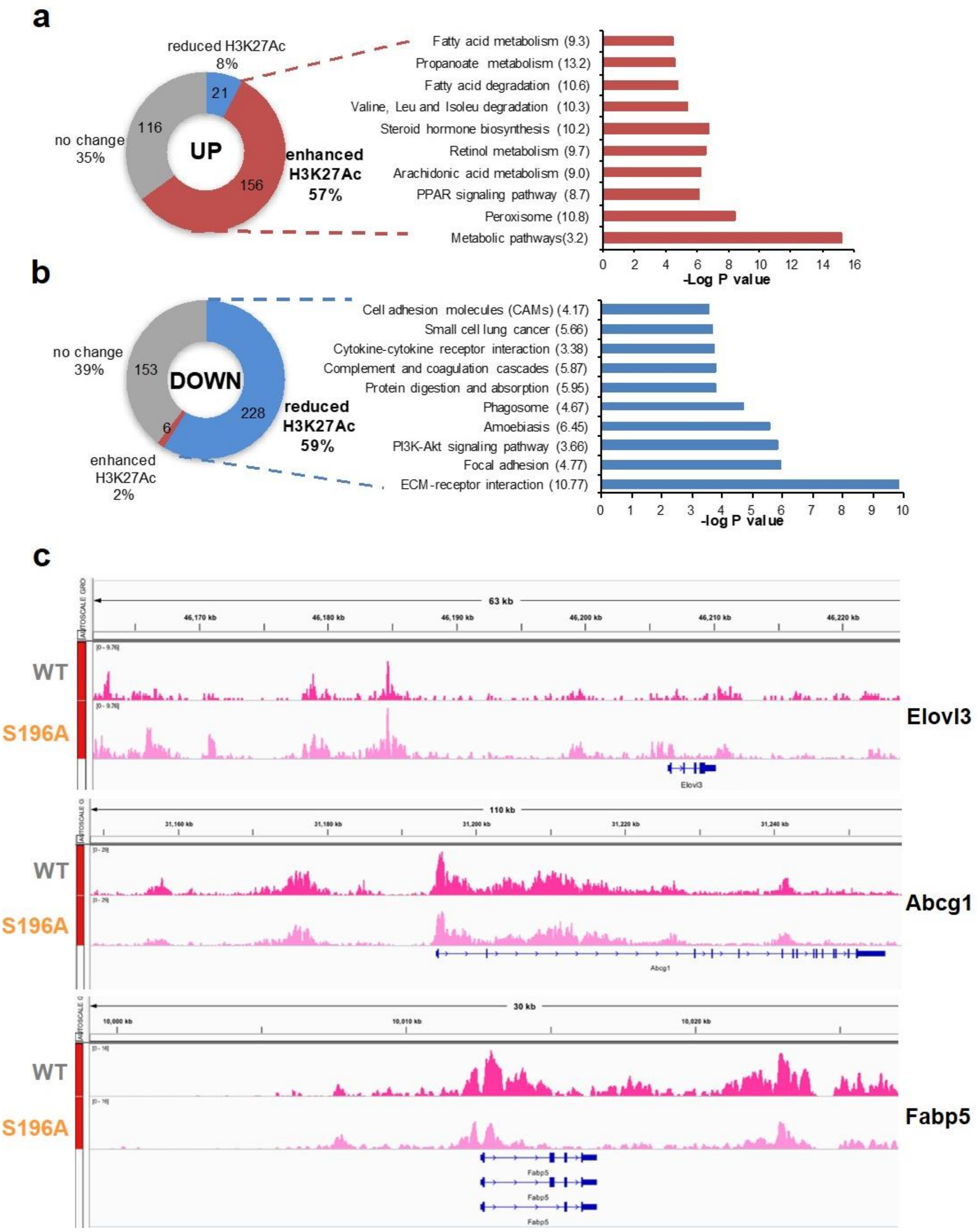

(a-b) Proportion of genes that are up-(a) or down- (b) regulated in S196A vs WT fatty livers showing changes in H3K27Ac by ChIPseq analysis. Number of genes are shown inside the graph. Bar graphs shows top ten KEGG Pathways (FDR<0.05) with enrichment scores between brackets. **(c)** Representative H3K27Ac ChIP-seq read alignment tracks in WT and S196A HFHC-fed livers for up-regulated (*Elovl3*) and down-regulated (*Abcg1* and *Fabp5*) genes in S196A livers.

Figure S6. Related to Figure 6.

a

VO	LXRα	S198A	Peptide
0	13	37	NCoR
0	4	22	TBLR1

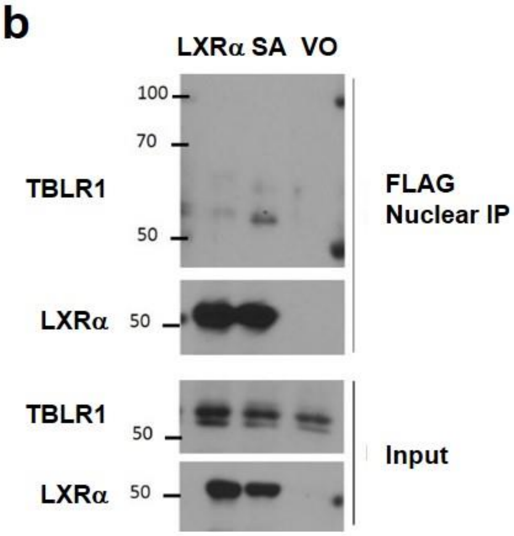

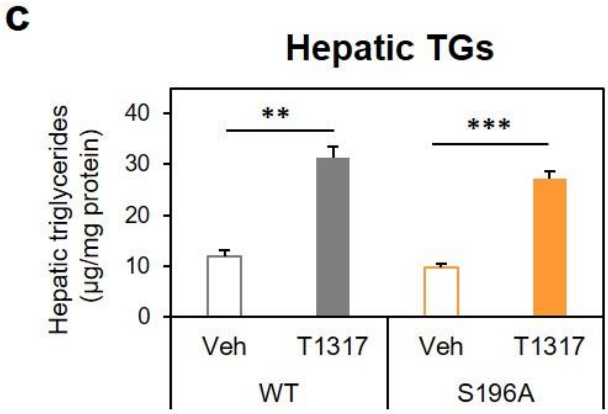

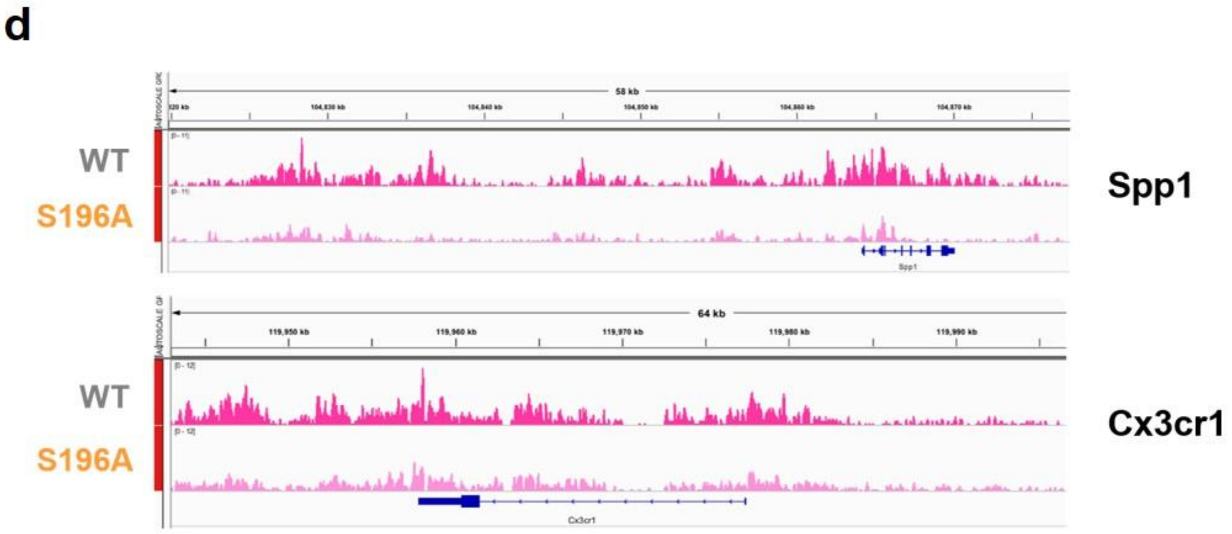

(a) Total spectral counts obtained from immunoprecipitates of wild-type human LXRα (LXRα), phospho-mutant (S198A) and control cells (expressing only the empty retroviral vector, VO) identified by mass spectroscopy. **(b)** Immunoprecipitation assays with cells expressing FLAG-tagged wild-type human LXRα (LXRα) and S198A (SA) mutant or vector only (VO). Wild-type and mutant LXRα were immunoprecipitated with anti-FLAG agarose beads followed by immunoblotting with specific TBLR1 antibodies. Expression of TBLR1 and LXRα in protein extracts prior to immunoprecipitation analysis (input) are shown. **(c)** Hepatic triglycerides (TGs) from WT and S196A mice treated with vehicle (Veh) or 50 mg/kg T0901317 (T1317). Values shown normalised to protein levels. **(d)** Representative H3K27Ac ChIP-seq read alignment tracks in WT and S196A HFHC-fed livers for genes shown to be reduced in S196A mice.

Table S1. Related to Figure 1.

Biometric and metabolic parameters of mice fed a chow diet.

Parameter	Genotype	Mean ± SEM	*p*
**Body weight (grams)**	WT	23.63 ± 0.6	0.132
	S196A	21.70 ± 0.75	
**% Liver weight (Liver g/Body g)**	WT	4.69 ± 0.25	0.241
	S196A	4.41 ± 0.07	
**Plasma glucose (mmol/L)**	WT	5.35 ± 0.10	0.268
	S196A	4.63 ± 0.22	
**Plasma insulin (ng/ mL)**	WT	0.34 ± 0.05	0.103
	S196A	0.87 ± 0.24	
**Hepatic triglycerides (µg / mg protein)**	WT	51.95 ± 5.06	0.116
	S196A	37.63 ± 4.50	
**Hepatic total cholesterol (µg / mg protein)**	WT	98.96 ± 10.48	0.688
	S196A	104.43 ± 4 .05	

Table S2. Related to Figure 1.

Biometric and metabolic parameters of mice fed a high fat and high cholesterol diet.

Parameter	Genotype	Mean ± SEM	p-value
**Body weight (grams)**	**WT**<br>**S196A**	21.36 ± 0 .41<br>19.89 ± 0.35	**0.012**
**% Liver weight (Liver g/Body g)**	**WT**<br>**S196A**	9.30 ± 0.17<br>6.41 ± 0.18	**3.06E-12**
**Plasma glucose (mmol/L)**	**WT**<br>**S196A**	4.49 ± 0.30<br>4.61 ± 0.24	0.762
**Plasma insulin (ng/ mL)**	**WT**<br>**S196A**	0.60 ± 0.10<br>0.87 ± 0.33	0.498
**Hepatic triglycerides (µg/ mg protein)**	**WT**<br>**S196A**	106.66 ± 10.04<br>170.41 ± 23.85	**0.033**
**Hepatic total cholesterol (µg/ mg protein)**	**WT**<br>**S196A**	207.79 ± 23.28<br>53.33 ± 2.76	**0.00006**

Table S3. Related to STAR Methods.

	**Forward primer** (5' to 3')	**Reverse primer** (5' to 3')
Adipophilin	GACCGTGCGGACTTGCTC	GCCATTTTTTCCTCCTGGAGA
Abca1	GGACATGCACAAGGTCCTGA	CAGAAAATCCTGGAGCTTCAAA
Abcg1	CCTTCCTCAGCATCATGCG	CCGATCCCAATGTGCGA
Abcg5	TGGATCCAACACCTCTATGCTAAA	GGCAGGTTTTCTCGATGAACTG
Abcg8	TGCCCACCTTCCACATGTC	ATGAAGCCGGCAGTAAGGTAGA
a-Sma	CCCAGACATCAGGGAGTAATGG	TCTATCGGATACTTCAGCGTCA
Atf3	GAGGATTTTGCTAACCTGACACC	TTGACGGTAACTGACTCCAGC
Atp6v0d2	GTGCAGTGTGAGACCTTGGA	GCCAGGAAGTTGCCATAGTC
Bex1	ATGGAGTCCAAAGATCAAGGCG	CTGGCTCCCTTCTGATGGTA
Cd36	GCCAAGCTATTGCGACATGA	TCTCAATGTCCGAGACTTTTCAAC
Ces1f	TGGAGAGTCAGCAGGAGGTT	ATGAAGGCCACACCACTCTC
Chop	CTGGAAGCCTGGTATGAGGAT	CAGGGTCAAGAGTAGTGAAGGT
Col1a1	GCTCCTCTTAGGGGCCACT	CCACGTCTCACCATTGGGG
Cyclophylin	GGCCGATGACGAGCCC	TGTCTTTGGAACTTTGTCTGCAA
Cyp17a1	ACCAGCCAGATCGGTTTATG	AGGGCAAATAACTGGGTGTG
Cyp2b13	ATGCTCATGTACCCCCATGT	GCCGATCACCTGATCAATCT
Cyp2b9	CTGGCCACCATGAAAGAGTT	CATTGGGCCTCCTCCTTTAT
Cyp2c69	CACAGTGGCTCATGAAGGAA	GATGAATTGGGGATCACAGG
Cyp7a1	CATACCTGGGCTGTGCTCTG	CCAAATGCC TTCGCAGAAGATG
Cx3cr1	TGAGTGACTGGCACTTCCTG	AAGGAGGTGGACATGGTGAG
Dgat2	CTGGCTGATAGCTGCTCTCTACTT	TGTGATCTCCTGCCACCTTTC
Elovl6	TGAACAAGCGAGCCAAGTTTG	GAGCACCGAATATACTGAAGACG
Fabp5	AGGATGGGAAGATGATCGTG	CTGGCAGCTAACTCCTGTCC
Fas	GCTGCGGAAACTTCAGGAAAT	AGAGACGTGTCACTCCTGGACTT
Fsp27	GTGTCCACTTGTGCCGTCTT	CTCGCTTGGTTGTCTTGATT
Hamp2	AGAAAGCAGGGCAGACATTG	GCAGATGGGGAAGTTGATGT
Idol	ATGCTGTGCTATGTCACGAGG	TCGATGATCCCTAGACGCCTG
Ldlr	GCATCAGCTTGGACAAGGTGT	GGGAACAGCCACCATTGTTG
L-Fabp	ATGAACTTCTCCGGCAAGTACC	CTGACACCCCCTTGATGTCC
Lpcat3	CCTTCACGGGCCTCTCAATT	CCATGAGTCGCAGGATGAGG
Lxra	GGTTGCTTTAGGGATAGGGTT	TTCCGCTTTTGTGGACGAAG
Nnmt	TGTGCAGAAAACGAGATCCTC	TGTGCAGAAAACGAGATCCTC
Ncp1l1	GAGAGCCAAAGATGCTACTATCTT	CCCGGGAAGTTGGTCATG
Osbpl3	AGACACGGAGGAGCACATCT	CGGTACATTCTGTGGTGACG
Osm	GCAGCTGTGGCTTTCTCTGG	TCGTCCCATTCCCTGAAGAC
Ppp1r3g	CTGAGACCCCGATCCCTGAT	GAGAGCGGCGATATTCCTGT
Scara3	GCTGGTGAAGACGAGGACAT	CAAAATCCGCACTGATGTGT
Scd1	CCGGAGACCCCTTAGATCGA	TAGCCTGTAAAAGATTTCTGCAAA
Slc22a26	ACAGAGCCCTGTATGGATGG	AGATCCACACACCAGGTTCC
Srebp1c	CAGGAGGACATC TTGCTGCTTC	TTGGGAGGCTGGTTTTGACC
Syngr1	CTGGTTCGTGGGTTTCTGCTT	GTCCCTTCGTTCAGAGGGTTG
Tgfb2	TTTGCTCCAGACAGTCCCAG	ATCTCCAGACATGCCAAGCC
Timp1	GTGGATATGCCCACAAGTCC	CTCAGAGTACGCCAGGGAAC
Thrsp	GCGGAAATACCAGGAAATGA	CGGGGTCTTCATCAGTCTTC
spl Xbp1	GAGTCCGCAGCAGGTG	GTGTCAGAGTCCATGGGA
Stk25	ACGTT CCTCCAACCATCCG	CTTCTGTGAGCTGTGCA
Wfdc3	CTTGGGTAGCTGCAGGAGAG	ATTCGTCTCCGGTACACAGC

Table S4. Related to STAR Methods.

	**Forward primer** (5' to 3')	**Reverse primer** (5' to 3')
Ces1f DR4	GGTGGTGGCCATTCAATATC	TGTCCACAAACCCTACCTGA
Ces1f TSS	CATTGACTTGGGAGCCTGTC	ACTCACCGCAAATCACACAG
Srebp1c LXRE	AGGCTCTTTTCGGGGATGG	TGGGGTTACTGGCGGTCAC
Srebp1c TSS	GTGGGCCTAGTCCGAAGC	ATCTCGGCCAGTGTCTGTTC
