# Supplementary material for: Impaired LXRα Phosphorylation Attenuates Progression of Fatty Liver Disease
Source: Cell Rep. 2019 Jan 22;26(4):984–995.e6. doi: 10.1016/j.celrep.2018.12.094 (PMC6344342; doi:10.1016/j.celrep.2018.12.094)
Supplement: Document S2. Article plus Supplemental Information [file mmc2.pdf]

Cell Reports

Article

Impaired LXRα Phosphorylation Attenuates Progression of Fatty Liver Disease

Graphical Abstract

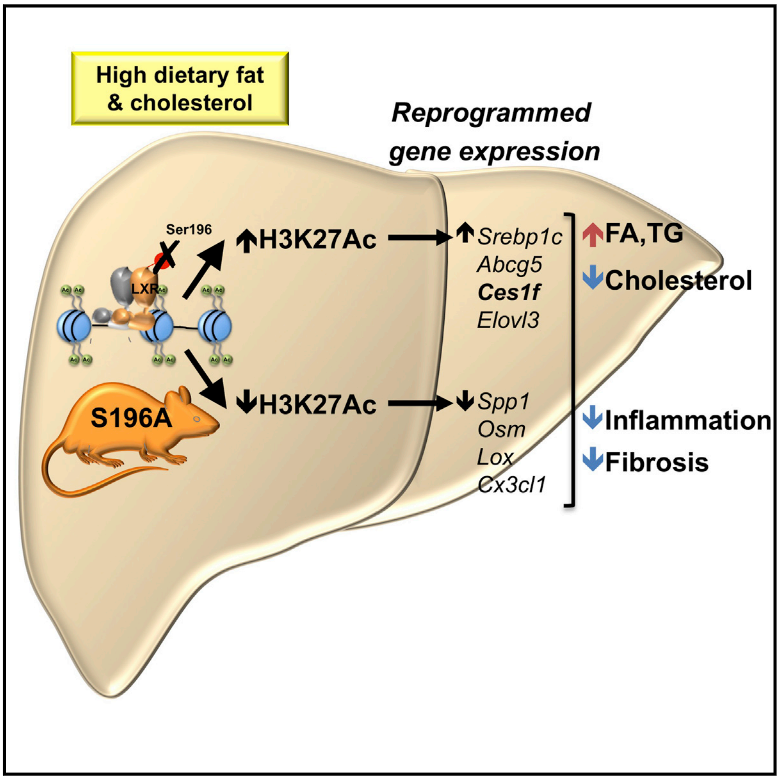

Highlights

- LXRαS196A induces liver steatosis and prevents cholesterol accumulation
- LXRαS196A reduces progression to hepatic inflammation and fibrosis
- LXRαS196A modulates hepatic chromatin acetylation
- LXRαS196A reveals unique dual LXRα phosphorylation and diet-responsive genes

Authors

Natalia Becares, Matthew C. Gage, Maud Voisin, ..., Krista Rombouts, Eckardt Treuter, Inés Pineda-Torra

Correspondence

i.torra@ucl.ac.uk

In Brief

Progression to inflammatory and fibrotic steatohepatitis is poorly understood. Becares et al. reveal that disrupting LXRα phosphorylation attenuates these processes by promoting a unique diet-induced transcriptome that prevents cholesterol accumulation and reduces hepatic inflammation and fibrosis. Mechanistically, phospho-deficient LXRα promotes chromatin modifications and alters protein-protein interactions in differentially expressed genes.

Becares et al., 2019, Cell Reports *26*, 984–995
January 22, 2019 © 2019 The Authors.
https://doi.org/10.1016/j.celrep.2018.12.094

CellPress

OPEN ACCESS
CellPress

Cell Reports
Article

Impaired LXRα Phosphorylation Attenuates Progression of Fatty Liver Disease

Natalia Becares,[1] Matthew C. Gage,[1] Maud Voisin,[2] Elina Shrestha,[2] Lucia Martin-Gutierrez,[1] Ning Liang,[3] Rikah Louie,[1] Benoit Pourcet,[1,9] Oscar M. Pello,[1,10] Tu Vinh Luong,[4] Saioa Goñi,[2,11] Cesar Pichardo-Almarza,[5] Hanne Røberg-Larsen,[6] Vanessa Diaz-Zuccarini,[5] Knut R. Steffensen,[7] Alastair O'Brien,[1] Michael J. Garabedian,[2] Krista Rombouts,[8] Eckardt Treuter,[3] and Inés Pineda-Torra[1,12,*]

[1]Centre of Cardiometabolic Medicine, Division of Medicine, University College of London, London WC1 E6JF, UK
[2]Department of Microbiology, New York University School of Medicine, New York, NY 10016, USA
[3]Karolinska Institute, Centre for Innovative Medicine (CIMED), Department of Biosciences and Nutrition, 14183 Huddinge, Sweden
[4]Department of Cellular Pathology, Royal Free London NHS Foundation Trust, London NW3 2QG, UK
[5]Department of Mechanical Engineering, University College London, London WC1E 7JE, UK
[6]Department of Chemistry, University of Oslo, 0371 Oslo, Norway
[7]Division of Clinical Chemistry, Department of Laboratory Medicine, Karolinska Institute, 14186 Huddinge, Sweden
[8]Institute for Liver & Digestive Health, University College London, Royal Free, London NW3 2PF, UK
[9]Present address: European Genomic Institute for Diabetes (EGID), FR 3508, Universite Lille, INSERM UMR 1011, Institut Pasteur de Lille, 59000 Lille, France
[10]Present address: HSCT and Cell Therapy Unit, Banco de Sangre y Tejidos de Cantabria, 39120 Liencres, Spain
[11]Present address: Program in Solid Tumors, Center for Applied Medical Research (CIMA) and Navarra's Health Research Institute (IDISNA), Pamplona, Spain
[12]Lead Contact
*Correspondence: i.torra@ucl.ac.uk
https://doi.org/10.1016/j.celrep.2018.12.094

SUMMARY

Non-alcoholic fatty liver disease (NAFLD) is a very common indication for liver transplantation. How fat-rich diets promote progression from fatty liver to more damaging inflammatory and fibrotic stages is poorly understood. Here, we show that disrupting phosphorylation at Ser196 (S196A) in the liver X receptor alpha (LXRα, NR1H3) retards NAFLD progression in mice on a high-fat-high-cholesterol diet. Mechanistically, this is explained by key histone acetylation (H3K27) and transcriptional changes in pro-fibrotic and pro-inflammatory genes. Furthermore, S196A-LXRα expression reveals the regulation of novel diet-specific LXRα-responsive genes, including the induction of Ces1f, implicated in the breakdown of hepatic lipids. This involves induced H3K27 acetylation and altered LXR and TBLR1 cofactor occupancy at the Ces1f gene in S196A fatty livers. Overall, impaired Ser196-LXRα phosphorylation acts as a novel nutritional molecular sensor that profoundly alters the hepatic H3K27 acetylome and transcriptome during NAFLD progression placing LXRα phosphorylation as an alternative anti-inflammatory or anti-fibrotic therapeutic target.

INTRODUCTION

Non-alcoholic fatty liver disease (NAFLD) is the major cause of chronic liver disease in the Western world affecting up to 30% of the adult population (70%–80% of obese and diabetics) and will become the main cause for liver transplantation by 2030 (European Association for the Study of the Liver (EASL); European Association for the Study of Diabetes (EASD); European Association for the Study of Obesity (EASO) et al., 2016). NAFLD involves conditions ranging from simple fatty liver accumulation or steatosis (triglyceride and cholesterol accumulation without significant alcohol consumption), steatosis accompanied by inflammation with or without fibrosis (steatohepatitis or NASH), progression to necrosis, cirrhosis, and hepatocellular carcinoma promoting liver-related mortality (European Association for the Study of the Liver (EASL); European Association for the Study of Diabetes (EASD); European Association for the Study of Obesity (EASO) et al., 2016). Steatosis alone is considered relatively benign, but its transition to NASH represents a key step into further liver damage, which without intervention can lead to organ transplantation. Despite its clinical relevance, the mechanisms underlying this transition are poorly understood. Indeed, there are currently no approved pharmacological interventions for NASH (Musso et al., 2012) and effective NAFLD therapies are restricted to weight loss through lifestyle modifications (Thoma et al., 2012). Therefore, identifying factors that modulate the transition to NASH is crucial for the development of treatments directly targeting NAFLD.

The Liver X receptors (LXRs) LXRα (Nr1h3) and LXRβ (Nr1h2) are key metabolic regulators. These lipid-activated transcription factors heterodimerize with the Retinoid X receptor (RXR) to control cholesterol and fatty acid homeostasis by regulating the expression of multiple enzymes, transporters, and modulators involved in these processes (Hong and Tontonoz, 2014). In addition, LXRs modulate inflammatory and immune pathways (Tall and Yvan-Charvet, 2015) and show anti-inflammatory and anti-fibrotic activities in experimental models of acute liver

 © 2019 The Authors.
This is an open access article under the CC BY license (http://creativecommons.org/licenses/by/4.0/).

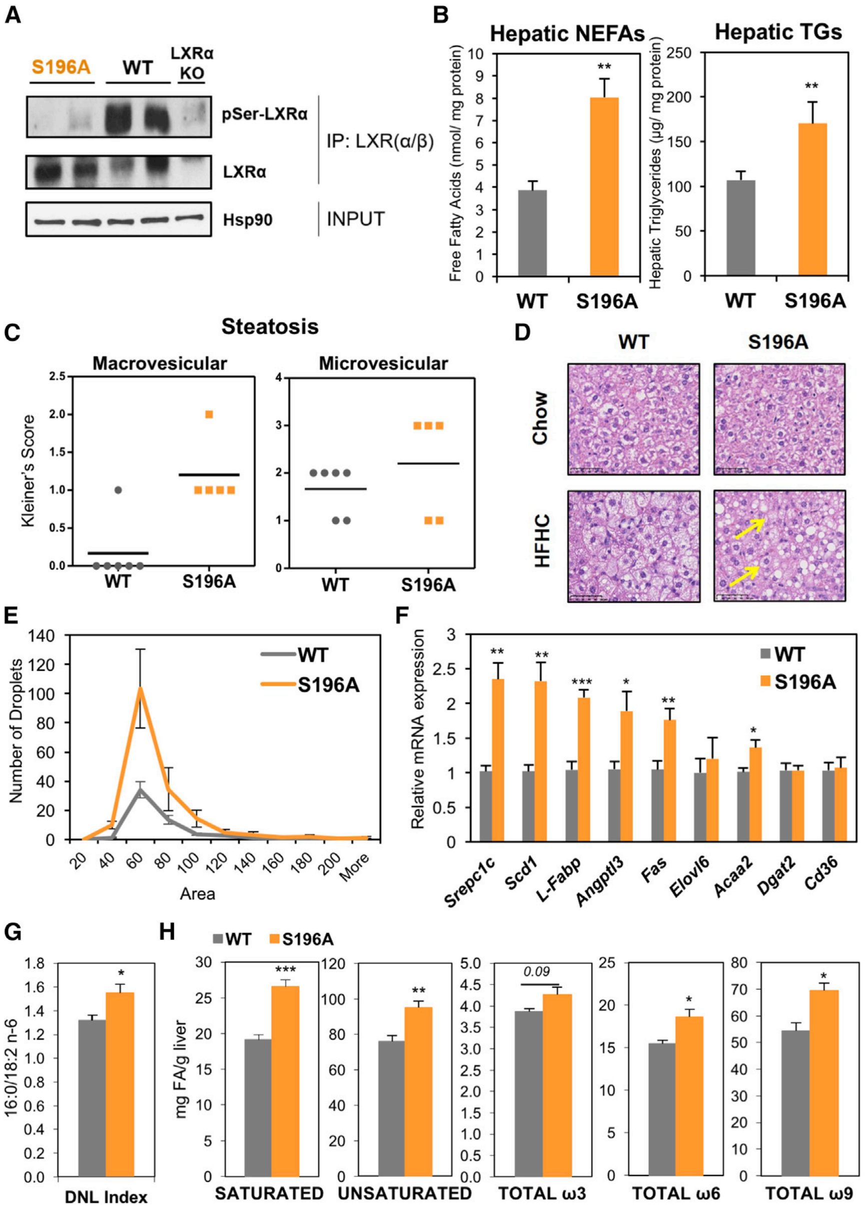

Figure 1. LXRα-S196A Mice Develop Enhanced Steatosis on a High-Cholesterol Diet

(A) LXRα-Ser196 phosphorylation analyzed by LXRα/β immunoprecipitation of liver homogenates and immunoblotting with a phospho-S196-LXRα-specific antibody. Global LXRα expression was assessed. Expression of Hsp90 as input loading control is shown.

(B) Hepatic non-esterified fatty acids (NEFAs) and triglycerides (TGs) in mice fed an HFHC diet (n = 6/group) normalized to liver protein levels.

(C) Kleiner's scores for steatosis (0–3) of liver sections (n $\geq$ 5/group).

(D) Representative images of H&E-stained liver sections from mice fed chow or HFHC diet. Arrows point at examples of microvesicular steatosis. Scale bar, 50 µM.

(E) Distribution of lipid droplets by area in H&E-stained liver sections (n = 6/group). Area distribution was compared by chi-square test for trend (p = 0.0003).

(F) Hepatic gene expression in mice fed an HFHC diet (n = 6/group). Normalized data are shown relative to WT, set as 1.

(G) *De novo* lipogenesis (DNL) index measured as the ratio of 16:0 (Palmitate) and 18:2 n-6 (Linoleic) content in liver (n = 6/group).

(H) Hepatic fatty acid levels (n = 6/group). Data are means ± SEM. $^*p < 0.05$, $^{**}p < 0.005$ or $^{***}p < 0.005$ relative to WT.

disease (Beaven et al., 2011; Hamilton et al., 2016). Besides ligand binding, LXR activity is regulated by post-translational modifications (Becares et al., 2017). We and others previously showed that LXRα is phosphorylated at Ser196 (Ser198 in the human homolog) (Chen et al., 2006; Torra et al., 2008; Wu et al., 2015) and that ligand-induced LXRα phosphorylation at this site alters its activity in a gene-specific manner in macrophage cell lines overexpressing the receptor (Torra et al., 2008; Wu et al., 2015). However, the physiological consequences of LXRα phosphorylation, and, specifically, the impact of disrupting LXRα phosphorylation on NAFLD progression, remain unknown. Here, we report that global LXRα phosphorylation at Ser196 as a nutritional sensor that critically impacts the transition to steatohepatitis in a dietary model of NAFLD. This could open much-needed alternative therapeutic avenues for NAFLD aimed at targeting this post-translational modification of LXRα.

RESULTS

LXRα-S196A Mice Exhibit Enhanced Steatosis

As we previously identified in macrophages (Torra et al., 2008; Wu et al., 2015), LXRα is phosphorylated at Ser196 (mouse) within a motif not present in LXRβ (Figure S1A), in both mouse (Figure 1A) and human liver (Figure S1B). To understand the impact of LXRα phosphorylation in response to a pathogenic diet, we generated a global knockin mouse carrying a homozygous serine-to-alanine mutation at Ser196 (S196A) that impairs its phosphorylation (Figures 1A and S1C–S1E) but does not affect overall hepatic LXRα levels (Figure S1F). On a chow diet, these mutant mice had no apparent dysmorphic phenotype and displayed similar developmental growth to matching wild-type (WT) mice (data not shown) and comparable hepatic lipids or other metabolic parameters (Table S1).

We previously found that cholesterol induces LXRα phosphorylation (Torra et al., 2008); thus, we hypothesized that LXRα phospho-mutant animals respond differently to a high-fat-high-cholesterol (HFHC) diet (Savard et al., 2013). Total body weight, plasma insulin, and glucose levels were similar between S196A and WT mice fed an HFHC diet (Table S2). By contrast, S196A

Table 1. LXRα Phosphorylation Alters Hepatic Fatty Acid Profiles

	WT	S196A	p Value
C16:1, c9	5.28 ± 0.401	6.85 ± 0.297	0.017*
C18:1, c9	48.69 ± 2.983	62.10 ± 2.631	0.012*
C18:1, c11	2.29 ± 0.136	2.93 ± 0.128	0.011*
C18:2, n-6	10.37 ± 0.409	13.11 ± 0.850	0.024*
C18:3, n-6	0.49 ± 0.028	0.66 ± 0.063	0.054
C18:3, n-3	0.39 ± 0.031	0.47 ± 0.043	0.205
C20:1, n-9	0.38 ± 0.014	0.51 ± 0.021	0.001*
C20:2, n-6	0.12 ± 0.004	0.14 ± 0.004	0.037*
C20:3, n-6	0.53 ± 0.028	0.44 ± 0.024	0.048*
C20:4, n-6	3.95 ± 0.081	4.27 ± 0.049	0.011*
C20:5, n-3	0.15 ± 0.006	0.16 ± 0.013	0.559
C22:5, n-3	0.17 ± 0.009	0.17 ± 0.010	0.589
C22:6, n-3	3.15 ± 0.055	3.45 ± 0.128	0.078

Hepatic fatty acid quantification in WT and S196A mice (n = 6/group). Data represent mean values of mg of fatty acid/g of liver tissue ± SEM. Significant differences ($p \leq 0.05$) are noted by an asterisk (*).

mice displayed higher levels of hepatic, but not plasma, non-esterified fatty acids (NEFAs) and triglycerides than WT mice (Figures 1B and S1F). Indeed, enhanced micro and macrovesicular hepatic steatosis, featuring more and larger lipid droplets, were observed in S196A liver sections (Figures 1C–1E) concomitant to enhanced expression of lipid droplet genes (Figure S1G). Additionally, increased steatosis was associated with enhanced hepatic expression of the lipogenic transcription factor sterol response element binding protein 1 (*Srebp1c*), and other known LXR target genes involved in fatty acid synthesis (fatty acid synthase, *Fas*) (Figure 1F). In contrast, other genes such as *Cd36,* involved in fatty acid uptake were not affected. Given that plasma NEFAs, triglycerides and insulin levels do not differ between genotypes (Figure S1F; Table S2), increased hepatic fat accumulation in S196A mice likely results from enhanced *de novo* lipogenesis (Figure 1G) as observed in other LXR models (Schultz et al., 2000). Notably, S196A mice showed an increase in the expression of stearoyl-CoA desaturase-1 (*Scd1*) (Figure 1F), which catalyzes the production of monounsaturated fatty acids. This led us to investigate whether changes in LXRα phosphorylation alter hepatic fatty acid composition, particularly since the saturation status of fatty acids accumulating in the liver during steatosis are thought to modulate the development of fatty liver and its progression to steatohepatitis (Peverill et al., 2014). Consistent with changes in gene expression, S196A livers showed an increase in the total amount of saturated as well as unsaturated fatty acids, specifically ω9 and certain ω6 fatty acid species (Figure 1H; Table 1). Altogether, our findings demonstrate that LXRα phosphorylation deficiency at S196 induces hepatic steatosis and alters fatty acid profiles in response to an HFHC diet.

Impaired LXRα Phosphorylation Attenuates Diet-Induced Hepatic Inflammation and Fibrosis

Diet-induced hepatic steatosis generally precedes inflammation and progression to fibrosis in experimental models (Sanyal, 2005). Strikingly, despite the increased steatosis, S196A mice displayed less inflammation (Figure 2A) and significantly less collagen deposition than their WT counterparts (Figure 2B). This was associated with a significant decrease in the expression of several pro-inflammatory and pro-fibrotic mediators, such as *Oncostatin M* (*Osm*), *Chemokine (C-X-C motif) ligand 1* (*Cxcl1*), and *Osteopontin* (*Spp1*) and genes involved in collagen synthesis (*Col1a1* and *Tgfb2*) (Figure 2C). Only a subset of the genes analyzed was affected by the S196A mutant (Figure 2C; data not shown) highlighting the gene-specific effects the mutant receptor has on its targets. Interestingly, reduced levels of inflammatory and fibrotic genes in S196A mice were revealed mostly upon exposure to the cholesterol-rich diet, while basal expression levels on chow were largely unaffected (Figure 2D). This likely reflects a modulatory role for LXRα phosphorylation in diet-induced transcriptional responses.

Pathways known to be implicated in the pathogenesis of lipid-induced liver damage, including apoptosis, lipid peroxidation, and macrophage content, were similar between genotypes (Figures S2A–S2C). In addition to these, prolonged adaptive endoplasmic reticulum (ER) stress is a known adaptive mechanism allowing cells to survive upon physiological changes requiring altered rates of protein folding. Notably, ER stress not only promotes steatosis, but also modulates hepatic fibrosis (Dara et al., 2011). Expression of factors involved in the activation of ER stress, such as the UPR target gene C/EBP homologous protein *(Chop*) and the Activating Transcription Factor (*Atf3*) or the spliced X-box-binding protein-1 (*Xbp-1*), was reduced in S196A mice (Figure 2E), suggesting these animals could be protected from lipotoxicity through a reduction in ER stress. Overall, these findings demonstrate that blocking LXRα-phosphorylation at S196 attenuates lipid-induced hepatic inflammation and fibrosis despite the observed enhanced steatosis by altering the expression of key molecules in these pathways.

LXRα Phospho-Mutant Mice Are Protected from Dietary Cholesterol Accumulation

Free cholesterol can act as a hepatotoxic agent (Marí et al., 2006) that induces collagen deposition in hepatic fibrosis (Tomita et al., 2014). In striking contrast to WT mice, S196A mice challenged with an HFHC diet were protected from plasma and hepatic cholesterol accumulation (Figure 3B). This was accompanied by a 20% reduction in liver weight in S196A livers compared to WT controls (Table S2), while hepatic bile acids remained unchanged (Figures S2D and S2E). Thus, we next investigated expression of genes involved in cholesterol metabolism pathways that could be altered by the LXRα phospho-mutant, some of which are already well-characterized targets of LXRα (Hong and Tontonoz, 2014). Reduced hepatic and plasma cholesterol levels were associated with decreased expression of cholesterol efflux transporter *Abcg1* (Figure 3C), reflecting a dampened response to the cholesterol-rich diet (Figure 3D). Notably, S196A mice showed a unique response to the HFHC diet regarding the upregulation of *Abcg5* (Figures 3C and 3D), a transporter mediating hepatobiliary cholesterol secretion (Wu et al., 2004) and a well-characterized target of LXRα (Hong and Tontonoz, 2014). No difference was observed in the levels of genes involved in bile acid synthesis (*Cyp7a1*, Figure 3C) or cholesterol intestinal absorption and

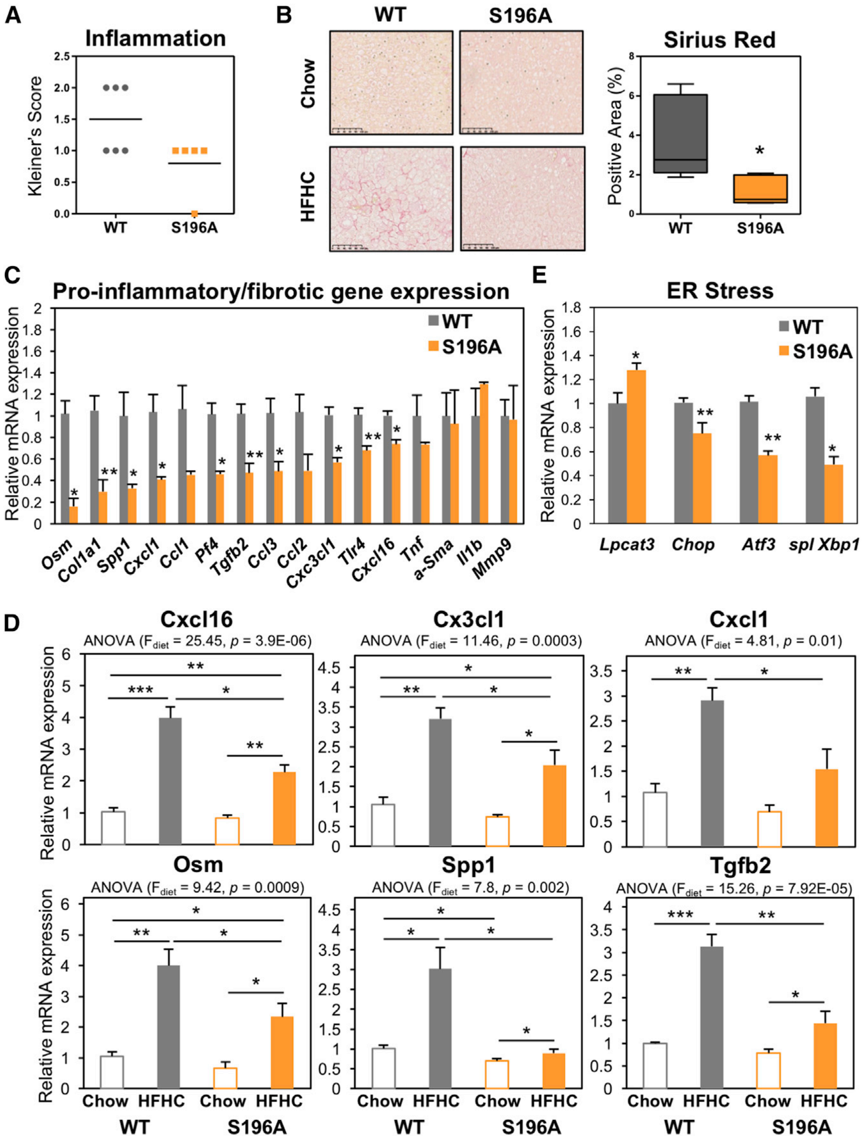

Figure 2. LXRα-S196A Alleviates Diet-Induced Hepatic Inflammation and Fibrosis

(A) Kleiner's scores for lobular inflammation (0–3) from liver sections of mice (n = 6/group).

(B) Representative images of Picrosirius-Red-stained liver sections (left). Scale bar, 100 μM. Quantification of stained areas by ImageJ (n = 6/group) (right). Values are the average of positively stained area.

(C) Hepatic gene expression (n = 6/group). Normalized data are shown relative to WT. Data are means ± SEM. *$p < 0.05$, **$p < 0.005$ relative to WT.

(D) Hepatic gene expression in mice fed chow (n = 4/group) or HFHC diet (n = 6/group). Normalized data are shown relative to WT chow group. *$p < 0.05$, **$p < 0.005$, ***$p < 0.0005$, relative to WT chow. Data are means ± SEM. *$p < 0.05$, **$p < 0.005$ relative to WT.

(E) Hepatic gene expression (n = 6/group). Values shown are normalized to cyclophilin and relative to WT.

excretion (Figure S2F), an important means by which LXR controls cholesterol homeostasis (Hong and Tontonoz, 2014), nor in the expression of other nuclear receptors regulating lipid metabolism (Figure S2G; data not shown). Thus, reduced cholesterol accumulation in S196A mice is likely due to increased hepatobiliary secretion of cholesterol. Moreover, in contrast to the strong repression of the cholesterogenic transcription factor *Srebp2* and its target gene *Ldlr* by dietary cholesterol in WT mice (Figures 3E and S2H), expression of these genes was largely unaffected by exposure to the diet in S196A mice, consistent with the unchanged hepatic cholesterol levels in these animals when challenged with the HFHC diet (Figure 3A). Overall, these differences in gene expression further reflect how the response to a cholesterol-rich diet differs between genotypes.

Intracellular cholesterol accumulation activates the unfolded protein response pathway in the ER (Devries-Seimon et al., 2005), inhibiting protein transport to the Golgi to re-establish ER function (Kockx et al., 2012). One gene linking cholesterol metabolism and ER stress is *Tm7sf2*, which not only participates in cholesterol biosynthesis as a 3β-hydroxysterol Δ14-reductase but also acts as an ER sensor by triggering anti-inflammatory pathways (Bellezza et al., 2013). Consistent with a decrease in hepatic inflammation (Figure 2A), *Tm7sf2* expression was enhanced in S196A mice exposed to the diet, while other genes involved in cholesterol biosynthesis were largely unaffected (Figure S2I). This suggests cholesterol modulation of ER stress responses, rather than cholesterol biosynthesis itself, is altered in S196A mice.

We next examined the levels of oxysterols, oxidized cholesterol derivatives some of which act as LXR ligands (Janowski et al., 1996), as these metabolites have been reported to be enhanced in NAFLD patients (Ikegami et al., 2012). Interestingly, LXRα phospho-mutant mice showed significantly reduced plasma levels of most oxysterols examined (Figure 3F), consistent with the reduced signs of hepatic inflammation and fibrosis (Figure 3C). Impaired oxysterol levels could be explained by the induced *Cyp7b1* expression (Figure 3C), an enzyme involved in oxysterol catabolism (Uppal et al., 2007), in S196A mice, while enzymes implicated in oxysterol synthesis remained unaffected (data not shown). Overall, these findings suggest that inhibition of LXRα phosphorylation acts as a molecular sensor of dietary cholesterol in the progression to steatohepatitis.

LXRα-S196A Reprograms Hepatic Gene Expression and Uncovers a Diet-Induced LXRα Transcriptome

To better understand the impact on diet-induced responses by the expression of the LXRα-S196A mutant and identify pathways sensitive to LXRα phosphorylation, we assessed global gene

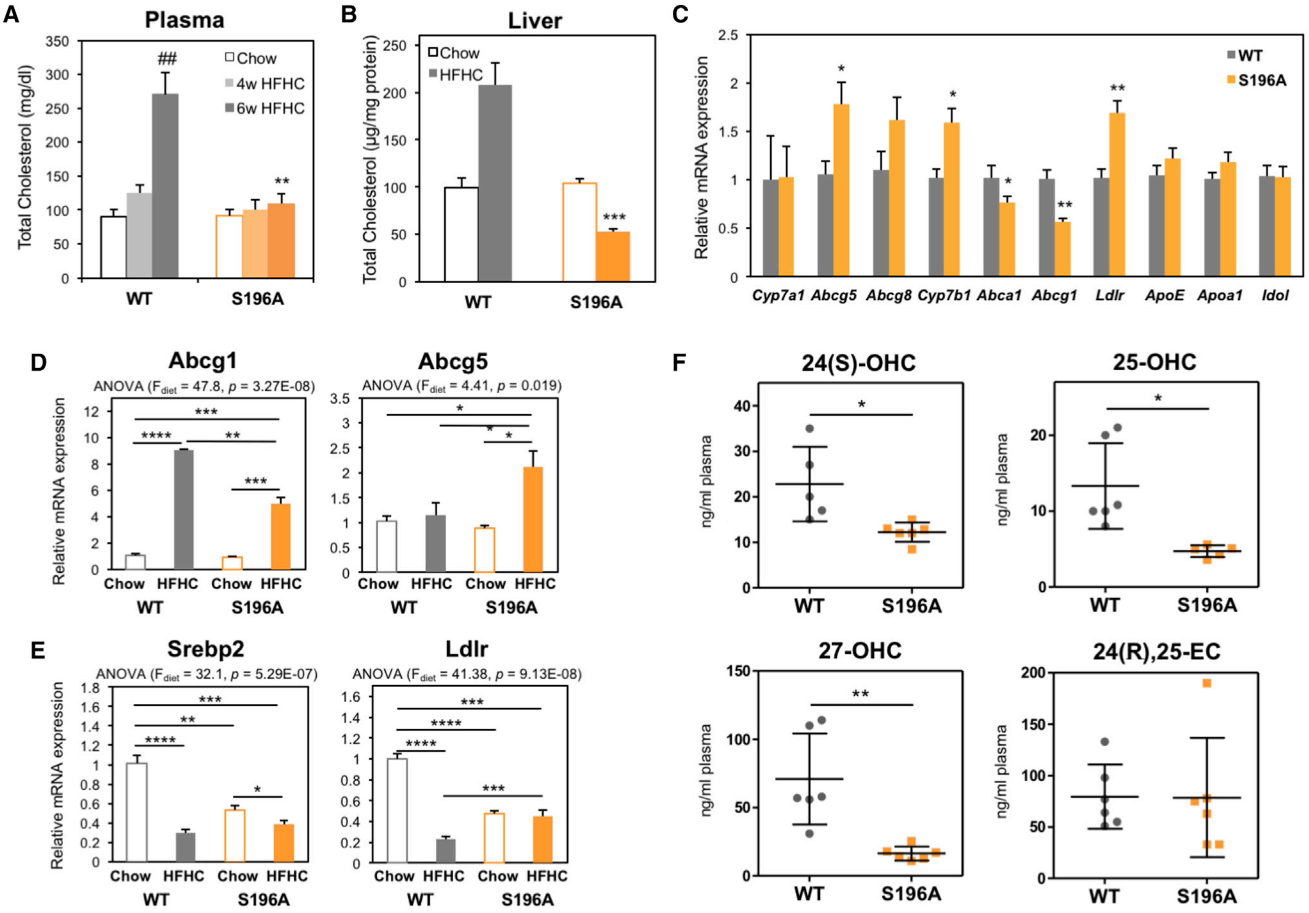

Figure 3. LXR Phosphorylation-Deficient Mice Show Reduced Cholesterol Levels in Response to an HFHC Diet

(A) Plasma total cholesterol levels in mice fed a chow (n = 4/group) or an HFHC diet (n ≥ 5/group).

(B) Hepatic total cholesterol levels in mice fed a chow (n = 4/group) or HFHC diet (n = 6/group). Values shown are normalized to protein levels in tissue homogenates.

(C) Hepatic gene expression in mice fed an HFHC diet (n = 6/group). Normalized data are shown relative to WT.

(D and E) Abcg1 and Abcg5 (D) and Srebp2 and Ldlr (E) hepatic gene expression in mice fed chow (n = 4) or an HFHC diet (n = 6). Normalized data are shown relative to WT chow group. Significance of comparisons between HFHC WT and S196A genotypes is shown in C).

(F) Quantification of free oxysterols in plasma of mice fed an HFHC diet (n = 6/group).

Data are means ± SEM. *p < 0.05, **p < 0.005, ***p < 0.0005, ****p < 0.00005 relative to WT. ##p < 0.005, 4 versus 6 weeks.

expression differences between WT and phospho-mutant genotypes by RNA sequencing (RNA-seq) analysis. Principal-component analysis evidenced that transcriptomes of WT and S196A mice are substantially different, especially under a cholesterol-rich diet (Figure 4A). Transcriptomic analysis revealed 668 genes whose hepatic expression is significantly altered in the mutant mice fed an HFHC diet (Figures 4B, 4C, and S3). Remarkably, there is minimal overlap between the genes modulated by LXRα phosphorylation at S196A in chow and HFHC diets, further reflecting on a genome-wide scale the distinct response exerted by S196A mice to the metabolic challenges posed by a cholesterol-rich diet. Pathway enrichment analysis confirmed our initial findings and showed induction of genes in lipid metabolism (Figures 4D and 4E). Further interrogation of our datasets revealed that, in addition to increased expression of enzymes involved in fatty acid synthesis (*Srebf1*, *Fas*; Figure 1F), S196A expression increased the hepatic levels of enzymes involved in fatty acid elongation (Elovl3, Elovl5) and fatty acid oxidation, with a trend toward increased levels of fatty acid desaturation enzyme *Fads1* (Figure 4F). These changes likely contribute to the distinct hepatic fatty acid profile of S196A mice (Figure 1H). Interestingly, expression of most of these enzymes is severely repressed by the HFHC diet (Figure S4A) further highlighting the modulatory role exerted by LXRα-S196A. Also corroborating previous analyses, the phospho-mutant mice showed a robust decrease in the levels of wound healing and fibrotic mediators, including several collagen genes and enzymes responsible for collagen stabilization (Figures 4D and 4G). Importantly, gene expression changes in response to an HFHC diet were substantially different in WT and S196A mice (Figures S4B–S4D), further indicating that impaired phosphorylation of LXRα-S196 alters the susceptibility to diet-induced hepatic injury by inducing a distinct hepatic

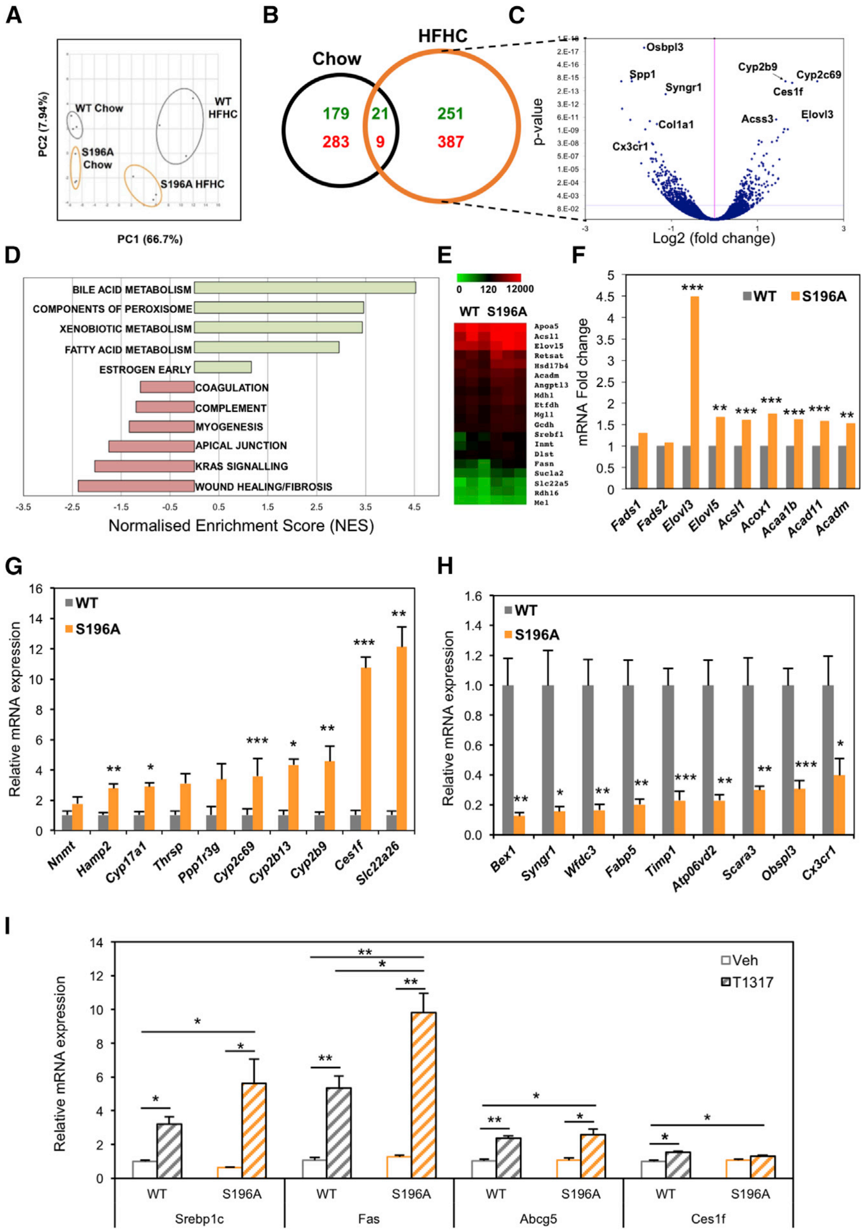

Figure 4. Changes in LXRα Phosphorylation Reprogram Hepatic Gene Expression

(A) Principal-component (PC) analysis plot showing RNA-seq samples analyzed by diet and genotype.

(B) Venn diagram of genes regulated by LXRαS196A compared to LXRαWT (±1.3-fold, $p < 0.05$) in the indicated diets. Numbers of upregulated and downregulated genes are depicted in green and red, respectively.

(C) Volcano plot of $\log_2$ ratio versus p value of differentially expressed genes comparing S196A and WT livers exposed to an HFHC (n = 3/group). Blue line indicates adjusted p value of 0.04 (Wald test for logistic regression).

(D) GSEA analysis showing enriched pathways in S196A livers with $p < 0.5$ (100 permutations) derived from HALLMARK gene sets.

(E) Heatmaps of hepatic RNA-seq raw gene counts (n = 3/genotype) for fatty acid and triglyceride metabolism.

(F) Fold change of hepatic RNA-seq gene counts in S196A compared to WT mice fed an HFHC diet (n = 3/genotype).

(G and H) Hepatic gene expression by qPCR of top (G) upregulated and (H) downregulated genes from the RNA-seq analysis on experimentally independent WT and S196A livers (n = 6/genotype).

(I) Hepatic gene expression from WT and S196A mice treated with vehicle or 50 mg/kg T0901317 (n = 4/group).

Data are normalized to cyclophilin and shown relative to WT vehicle set as 1. Significance was determined using single variance ANOVA followed by Student's t test. Normalized data are shown relative to WT as mean ± SEM. *$p < 0.05$, **$p < 0.005$ or ***$p < 0.005$

transcriptome. Moreover, expression of a subset of genes involved in extracellular matrix remodeling and tissue regeneration shown to distinguish between low-risk to mild and high-risk/severe NAFLD among pre-symptomatic patients (Moylan et al., 2014) was remarkably different between genotypes (Figure 4H). This suggests that changes in LXRα phosphorylation could alter pre-clinical NAFLD progression and emphasizes a role for Ser196-LXRα phosphorylation in the regulation of these remodeling pathways.

Genes showing the strongest difference in expression between WT and S196A genotypes were confirmed in a separate set of mice (Figures 4I and 4J). Notably, the majority of these genes were modulated by LXRα phosphorylation only in a cholesterol-rich environment (Figures S4E and S4F) and have not been previously reported to be regulated by LXR. One such gene, *Ces1f*, is a member of the carboxylesterase 1 family that hydrolyses cholesterol esters and triglycerides and controls hepatic lipid mobilization (Quiroga et al., 2012; Zhao et al., 2005). Despite previous studies showing *Ces1f* is not regulated by LXR ligands (Jones et al., 2013), we now clearly demonstrate *Ces1f* is highly sensitive to LXRα phosphorylation, preferentially in the context of an HFHC diet (Figures 4I, S4G, and S4H). To further demonstrate the lack of *Ces1f* regulation by the LXR ligand, we treated WT and S196A mice with the LXR synthetic agonist T0901317 and assessed changes in hepatic gene expression. While both genotypes clearly exhibited responses as exemplified by the increased expression of the traditional target genes *Srebp-1c* or *Fas* (Figure 4I) and hepatic triglycerides (Figure 6C), no differences were observed in the expression of *Ces1f* between ligand-treated WT and S196A mice. Moreover, expression of phosphorylation-sensitive genes, such as *Abcg5*, did not mirror that seen in HFHC-diet-fed animals, further confirming that the effects of S196A mutation in the hepatic transcriptome of HFHC-fed animals are specific to the dietary environment.

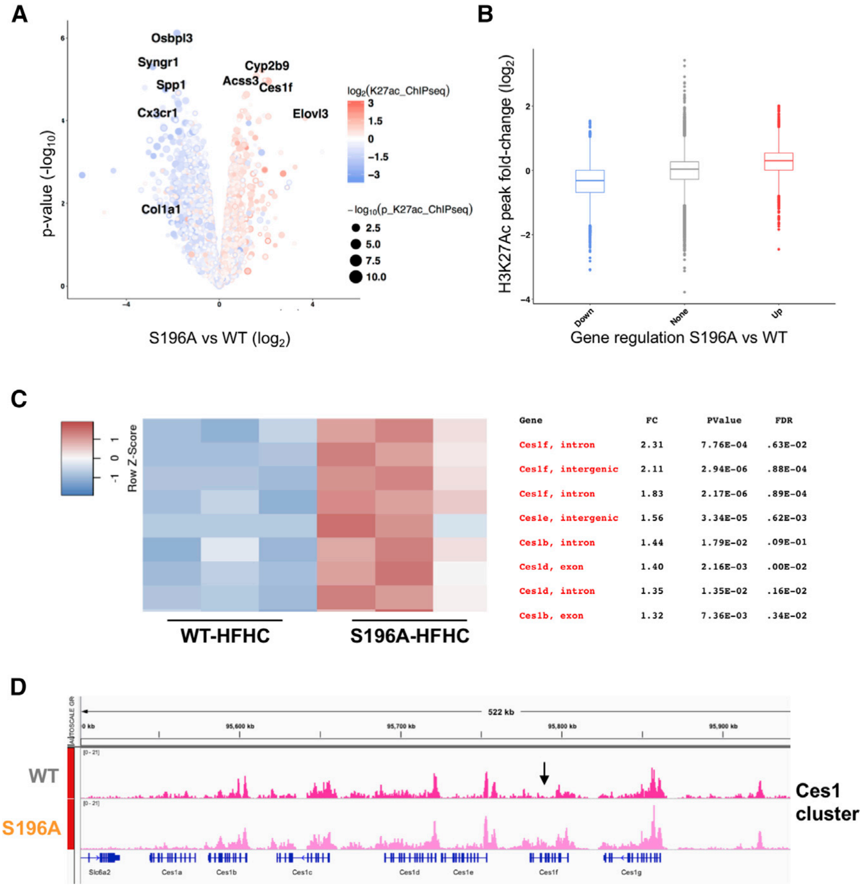

Figure 5. LXRα Phosphorylation at S196 Affects Global H3K27 Acetylation and LXR and TBLR1 Occupancy in the Ces1f Gene

(A) Volcano plot comparing differences in gene expression and H3K27Ac enriched sites shows $\log_2$ ratio versus $-\log_{10}$ p value of differentially expressed genes in S196A versus WT livers exposed to HFHC (n = 3). Colors show changes in H3K27Ac enrichment, and dot size depicts the p value.

(B) Boxplot showing the distribution of signal changes of altered H3K27Ac sites ($p < 0.05$) annotated to the upregulated (red), downregulated (blue), and unchanged (gray) genes in S196A versus WT livers by RNA-seq analysis.

(C) Heatmaps of H3K27Ac ChIP-seq counts (n = 3/genotype) for Ces family genes. Location, fold change (FC), p value, and false discovery rate (FDR) value are indicated for each peak.

(D) H3K27Ac ChIP-seq read alignment tracks in WT and S196A livers for Ces family gene cluster. The arrow marks location of identified DR4.

Altogether, these data highlight the relevance of LXRα phosphorylation in revealing LXR target genes and in modulating transcriptional responses to dietary cholesterol.

Identification of l LXR Binding Sites in Dual LXRα Phosphorylation and Diet-Sensitive Genes

We next investigated whether variation in the hepatic transcriptome observed in the S196A mice coincided with changes in chromatin modifications by chromatin immunoprecipitation sequencing (ChIP-seq) analysis. We specifically interrogated histone 3 lysine-27 acetylation (H3K27ac), a known marker for active regulatory elements in promoters and enhancers (Creyghton et al., 2010) previously shown to be modulated by a fat-rich diet (Siersbæk et al., 2017). Remarkably, differences in H3K27 acetylation between WT and S196A identified in a different set of animals strongly overlapped with changes in gene expression (Figures 5A and 5B). Indeed, most of the H3K27 acetylation observed at genes up- and downregulated by LXRαS196A was enhanced and reduced, respectively (Figures S5A–S5C). Collectively, these findings support the idea that the LXRα phospho-mutant affects gene expression partly through chromatin modifications.

To better understand the mechanism behind the differential gene regulation resulting from LXRαS196A expression, we next examined whether changes at LXRα phosphorylation at S196 affect LXR occupancy at selected genes in the context of a cholesterol-rich diet. We chose Ces1f as an example of a phosphorylation-sensitive target gene since its expression is strongly induced in S196A livers on an HFHC diet and its locus shows significant H3K27Ac enrichment (Figures 5A, 5C, and 5D). *In silico* analysis of the Ces1f gene locus showing enhanced H3K27ac enrichment identified a degenerated DR4 sequence (AGGTCTatttAGTTCA), resembling the consensus binding site or LXRE (Boergesen et al., 2012). This site was preferentially bound by LXR, but not its heterodimer partner RXR, in HFHC-fed S196A livers (Figure 6A). This was associated with increased RNA Polymerase II (Pol II) and phosho-Ser2 Pol II (pSer-Pol II) occupancy indicative of an enhanced transcriptional initiation and elongation, respectively, at the Ces1f locus (Figure 6C). In contrast, occupancy by both LXR and RXR to the well-established LXRE in Srebp-1c gene was induced (Figure 6B), as was Pol II and pSer-Pol II (Figure 6C). This shows impaired LXRαS196 phosphorylation allows the transcriptional activation of a gene containing degenerated DR4 sequences without affecting RXR occupancy.

This LXR binding sequence was revealed by homology to previously reported LXREs identified in response to LXR ligands in the context of a chow diet (Boergesen et al., 2012), as these are the only global LXR occupancy analyses available. However, ligand-induced responses may not necessarily phenocopy nuclear receptor and transcription factor binding patterns in response to a cholesterol-rich diet. Indeed, and in contrast to diet-fed animals (Figure 6A), WT and S196A female mice treated with T0901317 did not elicit differences in hepatic LXR occupancy at *Srebp-1c* LXRE and *Ces1f* DR4 sequences (Figure 6D). Moreover, pSer196-LXRα occupancy in chow and HFHC-fed livers of WT mice was reduced upon diet in the *Srebp-1c* LXRE region (Figure 6E), and a similar pattern was observed for global LXR occupancy (Figure 6F). Occupancy of the phospho-LXRα on

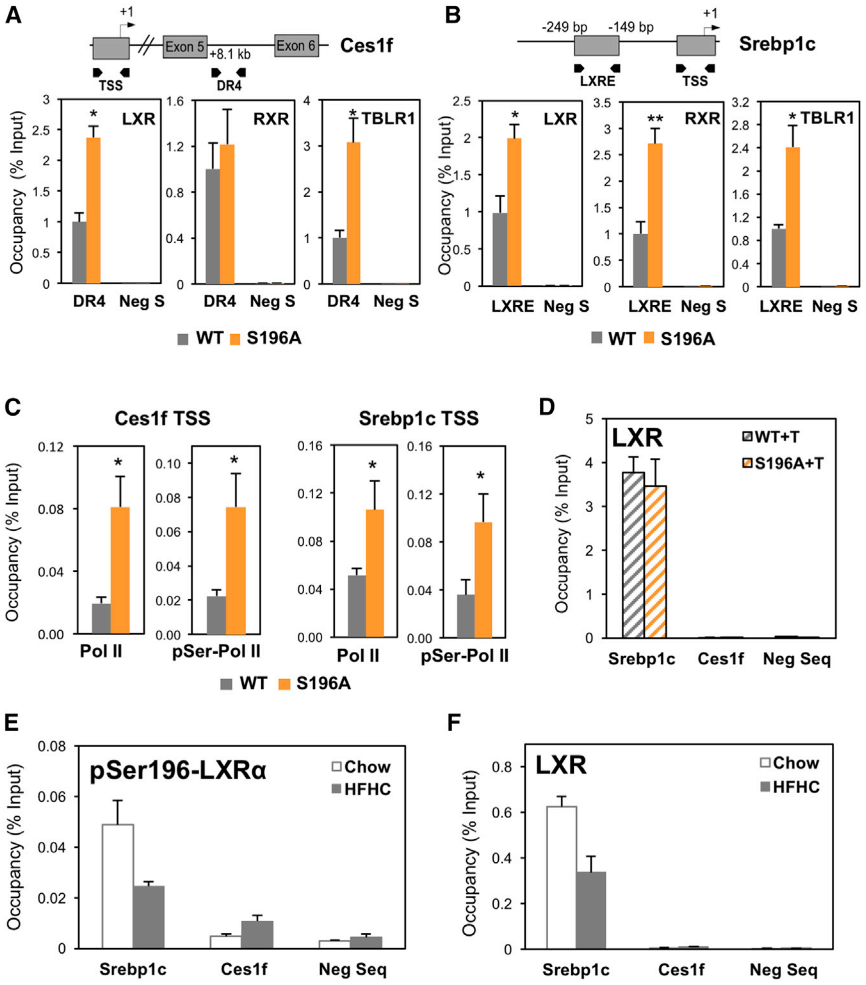

Figure 6. Identification of LXR Binding Sites in LXRα Phosphorylation/Diet-Sensitive Genes

(A and B) LXR, RXR, and TBLR1 occupancy at Ces1f newly identified DR4 (A) and Srebp-1c LXRE (B) sequences or a region within a gene desert (Neg S) in livers of WT and S196A mice fed an HFHC for 6 weeks ($n \geq 3$/group).

(C) RNA Pol II and pSer2-Pol II occupancy at Ces1f and Srebp-1c transcription start site (TSS) in livers of WT and S196A mice fed an HFHC diet ($n \geq 3$).

(D) LXR occupancy at Srebp-1c LXRE, Ces1f DR4, and non-specific negative sequences in livers of WT and S196A mice treated with T0901317 (+T) (n = 3/group).

(E and F) pSer196-LXRα (E) and LXR occupancy (F) at Srebp1c LXRE, Ces1f putative DR4 and non-specific negative sequences in livers of WT mice fed a chow or HFHC diet for 6 weeks (n = 3/group). Results are normalized to input values. For (A)–(C), results are normalized to input values and shown relative to WT, set as 1. Data represent means ± SEM. *$p < 0.05$, **$p < 0.005$ determined by Student's t test.

the *Ces1f* DR4 sequence was very low (Figure 6D), reinforcing the idea that binding of LXRα on this region is enhanced preferentially by the phospho-mutant version of the receptor.

Molecular modeling studies suggest that phosphorylation of LXRα at S198 (murine S196) induces a structural change in the hinge region of the receptor (Torra et al., 2008; Wu et al., 2015). Previously, we showed that, upon LXR ligand activation, phosphorylation affects the transcriptional activity of LXRα by modulating the binding of the NCoR corepressor to phospho-sensitive genes (Torra et al., 2008). We were unable to detect differences in NCoR occupancy in mice exposed to the HFHC diet (data not shown), suggesting responses to cholesterol *in vivo* involve other transcriptional players whose interaction with LXRα is sensitive to its phosphorylation status. One such factor is TBLR1, which participates in nuclear receptor cofactor exchange (Perissi et al., 2004) and modulates LXR target gene expression in hepatic cells (Jakobsson et al., 2009). TBLR1 was found to preferentially bind to LXRα-S196A (Figures S6A and S6B) and, consistently, its occupancy at the *Ces1f* DR4 sequences was significantly enhanced in S196A livers exposed to the HFHC diet (Figures 6A and 6B) suggesting this is an important component facilitating the transcription of this gene by the LXRα phospho-mutant in the context of a cholesterol-rich diet. Collectively, these data indicate that disrupting LXRα phosphorylation at Ser196 affects diet-induced responses in liver and reveals LXR target genes partly through differential occupancy of LXR and TBLR1.

DISCUSSION

Despite its clinical relevance, the transition between relatively benign fatty liver steatosis to inflammatory and fibrotic steatohepatitis remains poorly understood. The role of LXRα in promoting fatty acid and triglyceride accumulation is well established and has proved a major obstacle in the development of LXR ligands as therapeutics against metabolic and cardiovascular disorders (Hong and Tontonoz, 2014). Additionally, the hepatic anti-fibrotic and anti-inflammatory actions of LXRs in animal models of advanced fibrosis shed light into additional pathways these receptors modulate toward advanced steatohepatitis (Beaven et al., 2011; Wouters et al., 2010). Based on the idea of the beneficial effects of reversing hepatic lipid accumulation, pharmacological antagonism of LXRs has been proposed as an effective therapy against NAFLD. For instance, a liver-selective LXR inverse agonist SR9238 was shown to suppress hepatic fatty acid synthesis and lipid accumulation leading to alleviated hepatic inflammation and fibrosis in an obese rodent model (Griffett et al., 2013, 2015). However, it remained to be defined how LXR affects the transition to early fibrotic inflammatory stages of NAFLD in the context of an established fatty liver, which is more clinically relevant. Previous studies focused their efforts at examining changes in LXRα expression and reported induced levels of LXRα present in steatotic, inflammatory, and fibrotic livers (Ahn et al., 2014; Lima-Cabello et al., 2011). These could,

however, represent an adaptive or a maladaptive or pathogenic response to the ongoing cellular and molecular changes. Others studies, however, have shown that LXRα is the only nuclear receptor whose expression is unaffected during progression to steatohepatitis (Aguilar-Olivos et al., 2015). These contradictions highlight the need for further studies investigating how LXRs affect this chronic liver disease. We now propose that changes in LXRα phosphorylation play a crucial role in these transitional stages of NAFLD.

Posttranslational modifications are a powerful means by which the activity and function of nuclear receptors can be altered. However, despite the key importance of certain nuclear receptors in maintaining metabolic homeostasis, our understanding of how these modifications impact on metabolic diseases is scarce (Becares et al., 2017). Notably, the physiological consequences of LXRα phosphorylation, sumoylation, and acetylation have only been studied *in vitro* or non-specifically in animal models by pharmacologically or genetically altering the enzymes enhancing or inhibiting these modifications (Becares et al., 2017). To directly address the impact of LXRα phosphorylation on NAFLD progression, we have now generated a mouse model harboring an S196A mutation that disrupts LXRα phosphorylation at Ser196.

We report that disrupting Ser196-LXRα phosphorylation affects hepatic diet-induced responses by attenuating progression to steatohepatitis despite promoting lipid accumulation (Figure 6). Importantly, LXRα phosphorylation at this residue dictates transcriptional responses to an HFHC diet that promotes early stages of NAFLD. Supporting our previous data in macrophages (Gage et al., 2018; Torra et al., 2008), we now show the S196A-LXRα mutation affects hepatic transcriptional regulation in a gene-dependent manner, rather than conferring an overall gain or loss of function. Despite abundant triglyceride and NEFA accumulation, consistent with an increased *de novo* lipogenesis gene program (Figures 1 and S1), S196A mice exhibit significantly less hepatic inflammation and fibrosis than WT animals (Figure 2). This protective phenotype is associated with a dramatic reduction in hepatic and plasma cholesterol (Figure 3) and a robust repression of numerous pro-inflammatory and pro-fibrotic mediators, including eleven collagen species, Lysyl oxidase (LOX), and lysyl oxidase-like proteins (LOXLs) critical for collagen stabilization through irreversible crosslinking (Kanta, 2016; Liu et al., 2016) (Figures 2 and 4).

Impaired LXRα phosphorylation uncovers diet-specific and phosphorylation-sensitive genes, i.e., genes responsive to changes in LXRα phosphorylation, primarily in the context of a cholesterol-rich diet (Figures 4 and S4). Additionally, these genes have not been reported to be traditional ligand-modulated LXR targets suggesting the regulation of these identified genes does not simply phenocopy ligand-induced LXR activation. For instance, previous and our own studies failed to show *Ces1f* regulation by LXR ligands (Jones et al., 2013), whereas we now demonstrate *Ces1f* is highly sensitive to LXRα phosphorylation in early steatohepatitis (Figures 4I, S4G, and S4H). Ces1 has been recently shown to be protective from liver inflammation and injury (Xu et al., 2016), and its hepatic deficiency strongly increases susceptibility to cholesterol-driven liver injury (Li et al., 2017). However, the specific contribution by the carboxylesterase 1 family member *Ces1f* in NAFLD progression has not been addressed. In addition to *Ces1f*, other Ces1 members (*Ces1b, Ces1c, Ces1d, Ces1e*) are differentially regulated by the LXRα phospho-mutant, most of which are also only revealed to be sensitive to LXRα phosphorylation in a cholesterol-rich environment (Figure S4G). Interestingly, the form previously shown to be induced by LXR synthetic ligands in liver, *Ces2c* (Jones et al., 2013), does not vary in S196A mice regardless of the diet (Figures S4G and S4H), further indicating that LXRα phosphorylation-sensitive genes in response to diet are not necessarily regulated by LXR ligands and vice versa.

The diet-sensitive-induced expression of *Ces1f* by the LXRα phosphorylation mutant associated with differential binding of LXR and TBLR1 to binding sequences in this gene. Our transcriptomic analysis showed that TBLR1 is regulated neither by changes in LXRα phosphorylation nor by exposure to the cholesterol-rich diet. Interestingly, TBLR1 activity itself is subject to regulation by posttranslational modifications (Perissi et al., 2008). This further supports that changes in posttranslational modifications are a quick, reversible, and targeted way to regulate the transcriptional machinery. It is likely that a combination of interactions in addition to TBLR1 explain the overall phenotype observed. Future proteomic analysis of cofactor complexes affected by expression of the phosphorylation mutant in NAFLD livers will be needed to dissect these interactions in detail and their impact in disease progression. This is, however, beyond the scope of our current investigation.

It is important to note that hepatic LXR occupancy at gene regulatory sites has never been explored in a cholesterol-rich diet setting before but, rather, in the context of a chow diet (Boergesen et al., 2012; Heinz et al., 2010). Future work will be needed to establish genome-wide binding patterns of LXR and possibly other nuclear receptors important in the regulation of hepatic metabolism in the context of this pro-fibrotic diet. Interestingly, recent studies have established that altered metabolic states promote chromatin modifications both in animal models fed fat-rich diets and in obese and diabetic individuals (Leung et al., 2014, 2016; Yuan et al., 2014). Some of these modifications are thought to affect chromatin accessibility and are considered to act as a "metabolic imprint" that is able to alter metabolic disease risk, such as diabetes and NAFLD. However, it was recently shown that transcriptional changes in response to a fat-rich diet that induces obesity are not associated with chromatin accessibility measured as DNase sensitivity and rather occur at pre-established regulatory regions that show differential enrichment of H3K27Ac (Siersbæk et al., 2017). Our findings show that HFHC diet-induced changes in hepatic gene expression in S196A mice are associated with altered H3K27Ac levels, suggesting that the LXRα phosphorylation mutant takes advantage of the altered chromatin landscape to modulate dietary transcriptional responses. We have previously shown that cholesterol induces LXRα phosphorylation at S196 *in vitro* (Torra et al., 2008) and *in vivo*, in macrophages present in atherosclerotic mice on a fat-rich diet and in livers exposed to an HFHC diet (Torra et al., 2008; data not shown). Additional future investigations beyond the scope of this study will be needed to further dissect the signal(s) that promotes hepatic LXRα phosphorylation at this site in animals exposed to different diets.

Overall, LXRα phosphorylation at Ser196 acts as a molecular sensor in response to nutritional challenges thus promoting a unique diet-induced transcriptome that modulates metabolic, inflammatory, and fibrotic responses key in NAFLD progression. Understanding how this and other posttranslational modifications of LXRs are modulated and their impact on liver physiology could open alternative therapeutic avenues for NAFLD.

STAR★METHODS

Detailed methods are provided in the online version of this paper and include the following:

- KEY RESOURCES TABLE
- CONTACT FOR REAGENT AND RESOURCE SHARING
- EXPERIMENTAL MODEL AND SUBJECT DETAILS
  - Generation of S196A transgenic animal models
  - Animal husbandry
  - Culture of transfected HEK293T cells
  - Human liver tissue
- METHOD DETAILS
  - Diet and drug studies and tissue collection
  - Plasma and liver lipids
  - Plasma glucose and insulin
  - Hepatic fatty acid profiles
  - Hepatic bile acid quantification
  - Oxysterol LC-MS analysis
  - Faecal cholesterol quantification
  - Gene expression analysis
  - RNA sequencing studies
  - Western Blotting
  - Histopathological analysis
  - Lipid droplet identification
  - TUNEL staining
  - Lipid peroxidation quantification
  - LXRα proteomic analysis
  - Screening of potential LXREs
  - ChIP-qPCR and ChIP-sequencing
- QUANTIFICATION AND STATISTICAL ANALYSIS
- DATA AND SOFTWARE AVAILABILITY

SUPPLEMENTAL INFORMATION

Supplemental Information includes six figures and four tables and can be found with this article online at https://doi.org/10.1016/j.celrep.2018.12.094.

ACKNOWLEDGMENTS

We are grateful to Prof. Edward Fisher (NYU School of Medicine) and Dr. James Thorne (University of Leeds) for insightful discussions. We also thank Dr. Ruth Lovering (University College London) for her help with the bioinformatics analysis of the RNA-seq datasets and Dr. Annabel F. Valledor for insight on the LXR ligand experimental setup and pilot ligand experiments. This work was supported by a Medical Research Council New Investigator Grant G0801278 (I.P.-T.), British Heart Foundation Project Grants PG/13/10/30000 (I.P.-T.) and PG/16/87/32492 (M.C.G.), UCL Grand Challenges PhD Studentship (N.B. and I.P.-T.), Academy of Medical Sciences Newton Advanced Fellowship (I.P.-T.), Royal Free Charity (I.P.-T.), University of Oslo DIATECH@UiO initiative (H.R.-L.), the Centre for Innovative Medicine at the Karolinska Institute grant CIMED-2-391/2016 (E.T.), the Swedish Research Council grant VR-2016-01743 (E.T.), the Swedish Cancer Society grant CAN-2015/609 (E.T.), the Swedish Diabetes Foundation grant DIA2016-157 (E.T.), the Novo Nordisk Foundation grant NN2017-21086 (E.T.), and the European Union FP7 HEALTH program grant HUMAN-F5-2013-602757 (E.T.). M.J.G. and M.V. were supported by NIH R01HL117226. C.P.-A. and V.D.-Z. received support by the EPSRC grant (EP/L000296/1) and the Leverhulme Trust Senior Research Fellowship (RF-2015-482) and V.D.-Z. by the Wellcome/EPSRC Centre for Interventional and Surgical Sciences grant (203145Z/16/Z).

AUTHOR CONTRIBUTIONS

N.B. performed most experiments and data analysis and prepared figures. M.C.G. helped to establish mouse colonies and performed experiments and data interpretation. M.V. performed mice ligand studies and immunohistochemistry. L.M.-G. and R.L. performed qPCR and analyzed data. B.P. and O.M.P. helped establish mouse colonies. T.V.L. scored liver sections. S.G. and N.L. developed liver ChIP-seq protocols, N.L. performed H3K27ac ChIP-seq, and N.L., S.G., E.T., and I.P.-T. analyzed sequencing data. C.P.-A. and V.D.-Z. developed lipid droplet software. H.R.-L. performed oxysterol analyses. A.O.B., K.R.S., and K.R. provided materials. M.J.G. provided materials and data, and E.S. performed proteomic and immunoprecipitation analyses. K.R., M.J.G., and E.T. helped with data interpretation. N.B. and I.P.-T. designed experiments, interpreted data, and wrote the manuscript. I.P.-T. conceived the study, secured funding, and supervised all aspects of the work.

DECLARATION OF INTERESTS

C.P.-A. is an employee of Certara UK and was an employee of University College London at the time of the study.

Received: January 11, 2018
Revised: November 1, 2018
Accepted: December 20, 2018
Published: January 22, 2019

REFERENCES

Aguilar-Olivos, N.E., Carrillo-Córdova, D., Oria-Hernández, J., Sánchez-Valle, V., Ponciano-Rodríguez, G., Ramírez-Jaramillo, M., Chablé-Montero, F., Chávez-Tapia, N.C., Uribe, M., and Méndez-Sánchez, N. (2015). The nuclear receptor FXR, but not LXR, up-regulates bile acid transporter expression in non-alcoholic fatty liver disease. Ann. Hepatol. *14*, 487–493.

Ahn, S.B., Jang, K., Jun, D.W., Lee, B.H., and Shin, K.J. (2014). Expression of liver X receptor correlates with intrahepatic inflammation and fibrosis in patients with nonalcoholic fatty liver disease. Dig. Dis. Sci. *59*, 2975–2982.

Anders, S., and Huber, W. (2010). Differential expression analysis for sequence count data. Genome Biol. *11*, R106.

Babicki, S., Arndt, D., Marcu, A., Liang, Y., Grant, J.R., Maciejewski, A., and Wishart, D.S. (2016). Heatmapper: Web-enabled heat mapping for all. Nucleic Acids Res. *44*, W147–W153.

Beaven, S.W., Wroblewski, K., Wang, J., Hong, C., Bensinger, S., Tsukamoto, H., and Tontonoz, P. (2011). Liver X receptor signaling is a determinant of stellate cell activation and susceptibility to fibrotic liver disease. Gastroenterology *140*, 1052–1062.

Becares, N., Gage, M.C., and Pineda-Torra, I. (2017). posttranslational modifications of lipid-activated nuclear receptors: Focus on metabolism. Endocrinology *158*, 213–225.

Bellezza, I., Roberti, R., Gatticchi, L., Del Sordo, R., Rambotti, M.G., Marchetti, M.C., Sidoni, A., and Minelli, A. (2013). A novel role for Tm7sf2 gene in regulating TNFα expression. PLoS ONE *8*, e68017.

Boergesen, M., Pedersen, T.A., Gross, B., van Heeringen, S.J., Hagenbeek, D., Bindesbøll, C., Caron, S., Lalloyer, F., Steffensen, K.R., Nebb, H.I., et al. (2012). Genome-wide profiling of liver X receptor, retinoid X receptor, and peroxisome proliferator-activated receptor α in mouse liver reveals extensive sharing of binding sites. Mol. Cell. Biol. *32*, 852–867.

Chen, M., Bradley, M.N., Beaven, S.W., and Tontonoz, P. (2006). Phosphorylation of the liver X receptors. FEBS Lett. *580*, 4835–4841.

Chong, M.F.-F., Hodson, L., Bickerton, A.S., Roberts, R., Neville, M., Karpe, F., Frayn, K.N., and Fielding, B.A. (2008). Parallel activation of de novo lipogenesis and stearoyl-CoA desaturase activity after 3 d of high-carbohydrate feeding. Am. J. Clin. Nutr. *87*, 817–823.

Creyghton, M.P., Cheng, A.W., Welstead, G.G., Kooistra, T., Carey, B.W., Steine, E.J., Hanna, J., Lodato, M.A., Frampton, G.M., Sharp, P.A., et al. (2010). Histone H3K27ac separates active from poised enhancers and predicts developmental state. Proc. Natl. Acad. Sci. USA *107*, 21931–21936.

Dara, L., Ji, C., and Kaplowitz, N. (2011). The contribution of endoplasmic reticulum stress to liver diseases. Hepatology *53*, 1752–1763.

Devries-Seimon, T., Li, Y., Yao, P.M., Stone, E., Wang, Y., Davis, R.J., Flavell, R., and Tabas, I. (2005). Cholesterol-induced macrophage apoptosis requires ER stress pathways and engagement of the type A scavenger receptor. J. Cell Biol. *171*, 61–73.

Duda, R.O., and Hart, P.E. (1972). Use of the Hough transformation to detect lines and curves in pictures. Commun. ACM *15*, 11–15.

European Association for the Study of the Liver (EASL); European Association for the Study of Diabetes (EASD); European Association for the Study of Obesity (EASO) (2016). EASL-EASD-EASO Clinical Practice Guidelines for the management of non-alcoholic fatty liver disease. J. Hepatol. *64*, 1388–1402.

Fan, R., Toubal, A., Goñi, S., Drareni, K., Huang, Z., Alzaid, F., Ballaire, R., Ancel, P., Liang, N., Damdimopoulos, A., et al. (2016). Loss of the co-repressor GPS2 sensitizes macrophage activation upon metabolic stress induced by obesity and type 2 diabetes. Nat. Med. *22*, 780–791.

Gage, M.C., Bécares, N., Louie, R., Waddington, K.E., Zhang, Y., Tittanegro, T.H., Rodríguez-Lorenzo, S., Jathanna, A., Pourcet, B., Pello, O.M., et al. (2018). Disrupting LXRα phosphorylation promotes FoxM1 expression and modulates atherosclerosis by inducing macrophage proliferation. Proc. Natl. Acad. Sci. USA *115*, E6556–E6565.

Griffett, K., Solt, L.A., El-Gendy, Bel.-D., Kamenecka, T.M., and Burris, T.P. (2013). A liver-selective LXR inverse agonist that suppresses hepatic steatosis. ACS Chem. Biol. *8*, 559–567.

Griffett, K., Welch, R.D., Flaveny, C.A., Kolar, G.R., Neuschwander-Tetri, B.A., and Burris, T.P. (2015). The LXR inverse agonist SR9238 suppresses fibrosis in a model of non-alcoholic steatohepatitis. Mol. Metab. *4*, 353–357.

Hamilton, J.P., Koganti, L., Muchenditsi, A., Pendyala, V.S., Huso, D., Hankin, J., Murphy, R.C., Huster, D., Merle, U., Mangels, C., et al. (2016). Activation of liver X receptor/retinoid X receptor pathway ameliorates liver disease in Atp7B(-/-) (Wilson disease) mice. Hepatology *63*, 1828–1841.

Heinz, S., Benner, C., Spann, N., Bertolino, E., Lin, Y.C., Laslo, P., Cheng, J.X., Murre, C., Singh, H., and Glass, C.K. (2010). Simple combinations of lineage-determining transcription factors prime cis-regulatory elements required for macrophage and B cell identities. Mol. Cell *38*, 576–589.

Hong, C., and Tontonoz, P. (2014). Liver X receptors in lipid metabolism: opportunities for drug discovery. Nat. Rev. Drug Discov. *13*, 433–444.

Howe, E.A., Sinha, R., Schlauch, D., and Quackenbush, J. (2011). RNA-Seq analysis in MeV. Bioinformatics *27*, 3209–3210.

Ikegami, T., Hyogo, H., Honda, A., Miyazaki, T., Tokushige, K., Hashimoto, E., Inui, K., Matsuzaki, Y., and Tazuma, S. (2012). Increased serum liver X receptor ligand oxysterols in patients with non-alcoholic fatty liver disease. J. Gastroenterol. *47*, 1257–1266.

Jakobsson, T., Venteclef, N., Toresson, G., Damdimopoulos, A.E., Ehrlund, A., Lou, X., Sanyal, S., Steffensen, K.R., Gustafsson, J.-Å., and Treuter, E. (2009). GPS2 is required for cholesterol efflux by triggering histone demethylation, LXR recruitment, and coregulator assembly at the ABCG1 locus. Mol. Cell *34*, 510–518.

Janowski, B.A., Willy, P.J., Devi, T.R., Falck, J.R., and Mangelsdorf, D.J. (1996). An oxysterol signalling pathway mediated by the nuclear receptor LXR alpha. Nature *383*, 728–731.

Jones, R.D., Taylor, A.M., Tong, E.Y., and Repa, J.J. (2013). Carboxylesterases are uniquely expressed among tissues and regulated by nuclear hormone receptors in the mouse. Drug Metab. Dispos. *41*, 40–49.

Kanta, J. (2016). Elastin in the Liver. Front. Physiol. *7*, 491.

Kockx, M., Dinnes, D.L., Huang, K.-Y., Sharpe, L.J., Jessup, W., Brown, A.J., and Kritharides, L. (2012). Cholesterol accumulation inhibits ER to Golgi transport and protein secretion: studies of apolipoprotein E and VSVGt. Biochem. J. *447*, 51–60.

Koentgen, F., Suess, G., and Naf, D. (2010). Engineering the mouse genome to model human disease for drug discovery. Methods Mol. Biol. *602*, 55–77.

Leung, A., Parks, B.W., Du, J., Trac, C., Setten, R., Chen, Y., Brown, K., Lusis, A.J., Natarajan, R., and Schones, D.E. (2014). Open chromatin profiling in mice livers reveals unique chromatin variations induced by high fat diet. J. Biol. Chem. *289*, 23557–23567.

Leung, A., Trac, C., Du, J., Natarajan, R., and Schones, D.E. (2016). Persistent chromatin modifications induced by high fat diet. J. Biol. Chem. *291*, 10446–10455.

Li, J., Wang, Y., Matye, D.J., Chavan, H., Krishnamurthy, P., Li, F., and Li, T. (2017). Sortilin 1 modulates hepatic cholesterol lipotoxicity in mice via Functional Interaction with liver carboxylesterase 1. J. Biol. Chem. *292*, 146–160.

Liang, W., Menke, A.L., Driessen, A., Koek, G.H., Lindeman, J.H., Stoop, R., Havekes, L.M., Kleemann, R., and van den Hoek, A.M. (2014). Establishment of a general NAFLD scoring system for rodent models and comparison to human liver pathology. PLoS ONE *9*, e115922.

Lima-Cabello, E., García-Mediavilla, M.V., Miquilena-Colina, M.E., Vargas-Castrillón, J., Lozano-Rodríguez, T., Fernández-Bermejo, M., Olcoz, J.L., González-Gallego, J., García-Monzón, C., and Sánchez-Campos, S. (2011). Enhanced expression of pro-inflammatory mediators and liver X-receptor-regulated lipogenic genes in non-alcoholic fatty liver disease and hepatitis C. Clin. Sci. (Lond.) *120*, 239–250.

Liu, S.B., Ikenaga, N., Peng, Z.-W., Sverdlov, D.Y., Greenstein, A., Smith, V., Schuppan, D., and Popov, Y. (2016). Lysyl oxidase activity contributes to collagen stabilization during liver fibrosis progression and limits spontaneous fibrosis reversal in mice. FASEB J. *30*, 1599–1609.

Marí, M., Caballero, F., Colell, A., Morales, A., Caballeria, J., Fernandez, A., Enrich, C., Fernandez-Checa, J.C., and García-Ruiz, C. (2006). Mitochondrial free cholesterol loading sensitizes to TNF- and Fas-mediated steatohepatitis. Cell Metab. *4*, 185–198.

Moylan, C.A., Pang, H., Dellinger, A., Suzuki, A., Garrett, M.E., Guy, C.D., Murphy, S.K., Ashley-Koch, A.E., Choi, S.S., Michelotti, G.A., et al. (2014). Hepatic gene expression profiles differentiate presymptomatic patients with mild versus severe nonalcoholic fatty liver disease. Hepatology *59*, 471–482.

Musso, G., Cassader, M., Rosina, F., and Gambino, R. (2012). Impact of current treatments on liver disease, glucose metabolism and cardiovascular risk in non-alcoholic fatty liver disease (NAFLD): A systematic review and meta-analysis of randomised trials. Diabetologia *55*, 885–904.

Pehkonen, P., Welter-Stahl, L., Diwo, J., Ryynänen, J., Wienecke-Baldacchino, A., Heikkinen, S., Treuter, E., Steffensen, K.R., and Carlberg, C. (2012). Genome-wide landscape of liver X receptor chromatin binding and gene regulation in human macrophages. BMC Genomics *13*, 50.

Perissi, V., Aggarwal, A., Glass, C.K., Rose, D.W., and Rosenfeld, M.G. (2004). A corepressor/coactivator exchange complex required for transcriptional activation by nuclear receptors and other regulated transcription factors. Cell *116*, 511–526.

Perissi, V., Scafoglio, C., Zhang, J., Ohgi, K.A., Rose, D.W., Glass, C.K., and Rosenfeld, M.G. (2008). TBL1 and TBLR1 phosphorylation on regulated gene promoters overcomes dual CtBP and NCoR/SMRT transcriptional repression checkpoints. Mol. Cell *29*, 755–766.

Peverill, W., Powell, L.W., and Skoien, R. (2014). Evolving concepts in the pathogenesis of NASH: beyond steatosis and inflammation. Int. J. Mol. Sci. *15*, 8591–8638.

Pourcet, B., Gage, M.C., León, T.E., Waddington, K.E., Pello, O.M., Steffensen, K.R., Castrillo, A., Valledor, A.F., and Pineda-Torra, I. (2016). The nuclear

receptor LXR modulates interleukin-18 levels in macrophages through multiple mechanisms. Sci. Rep. *6*, 25481.

Quiroga, A.D., Li, L., Trötzmüller, M., Nelson, R., Proctor, S.D., Köfeler, H., and Lehner, R. (2012). Deficiency of carboxylesterase 1/esterase-x results in obesity, hepatic steatosis, and hyperlipidemia. Hepatology *56*, 2188–2198.

Reimand, J., Arak, T., Adler, P., Kolberg, L., Reisberg, S., Peterson, H., and Vilo, J. (2016). g:Profiler-a web server for functional interpretation of gene lists (2016 update). Nucleic Acids Res. *44*, W83–W89.

Roberg-Larsen, H., Lund, K., Vehus, T., Solberg, N., Vesterdal, C., Misaghian, D., Olsen, P.A., Krauss, S., Wilson, S.R., and Lundanes, E. (2014). Highly automated nano-LC/MS-based approach for thousand cell-scale quantification of side chain-hydroxylated oxysterols. J. Lipid Res. *55*, 1531–1536.

Roberg-Larsen, H., Lund, K., Seterdal, K.E., Solheim, S., Vehus, T., Solberg, N., Krauss, S., Lundanes, E., and Wilson, S.R. (2017). Mass spectrometric detection of 27-hydroxycholesterol in breast cancer exosomes. J. Steroid Biochem. Mol. Biol. *169*, 22–28.

Robinson, J.T., Thorvaldsdóttir, H., Winckler, W., Guttman, M., Lander, E.S., Getz, G., and Mesirov, J.P. (2011). Integrative genomics viewer. Nat. Biotechnol. *29*, 24–26.

Sandelin, A., and Wasserman, W.W. (2005). Prediction of nuclear hormone receptor response elements. Mol. Endocrinol. *19*, 595–606.

Sanyal, A.J. (2005). Mechanisms of Disease: pathogenesis of nonalcoholic fatty liver disease. Nat. Clin. Pract. Gastroenterol. Hepatol. *2*, 46–53.

Savard, C., Tartaglione, E.V., Kuver, R., Haigh, W.G., Farrell, G.C., Subramanian, S., Chait, A., Yeh, M.M., Quinn, L.S., and Ioannou, G.N. (2013). Synergistic interaction of dietary cholesterol and dietary fat in inducing experimental steatohepatitis. Hepatology *57*, 81–92.

Schultz, J.R., Tu, H., Luk, A., Repa, J.J., Medina, J.C., Li, L., Schwendner, S., Wang, S., Thoolen, M., Mangelsdorf, D.J., et al. (2000). Role of LXRs in control of lipogenesis. Genes Dev. *14*, 2831–2838.

Shrestha, E., Hussein, M.A., Savas, J.N., Ouimet, M., Barrett, T.J., Leone, S., Yates, J.R., 3rd, Moore, K.J., Fisher, E.A., and Garabedian, M.J. (2016). Poly(ADP-ribose) polymerase 1 represses liver X receptor-mediated ABCA1 expression and cholesterol efflux in macrophages. J. Biol. Chem. *291*, 11172–11184.

Siersbæk, M., Varticovski, L., Yang, S., Baek, S., Nielsen, R., Mandrup, S., Hager, G.L., Chung, J.H., and Grøntved, L. (2017). High fat diet-induced changes of mouse hepatic transcription and enhancer activity can be reversed by subsequent weight loss. Sci. Rep. *7*, 40220.

Subramanian, A., Tamayo, P., Mootha, V.K., Mukherjee, S., Ebert, B.L., Gillette, M.A., Paulovich, A., Pomeroy, S.L., Golub, T.R., Lander, E.S., and Mesirov, J.P. (2005). Gene set enrichment analysis: a knowledge-based approach for interpreting genome-wide expression profiles. Proc. Natl. Acad. Sci. USA *102*, 15545–15550.

Takeuchi, T., Nomura, T., Tsujita, M., Suzuki, M., Fuse, T., Mori, H., and Mishina, M. (2002). Flp recombinase transgenic mice of C57BL/6 strain for conditional gene targeting. Biochem. Biophys. Res. Commun. *293*, 953–957.

Tall, A.R., and Yvan-Charvet, L. (2015). Cholesterol, inflammation and innate immunity. Nat. Rev. Immunol. *15*, 104–116.

Thoma, C., Day, C.P., and Trenell, M.I. (2012). Lifestyle interventions for the treatment of non-alcoholic fatty liver disease in adults: a systematic review. J. Hepatol. *56*, 255–266.

Tomita, K., Teratani, T., Suzuki, T., Shimizu, M., Sato, H., Narimatsu, K., Okada, Y., Kurihara, C., Irie, R., Yokoyama, H., et al. (2014). Free cholesterol accumulation in hepatic stellate cells: mechanism of liver fibrosis aggravation in nonalcoholic steatohepatitis in mice. Hepatology *59*, 154–169.

Torra, I.P., Ismaili, N., Feig, J.E., Xu, C.-F., Cavasotto, C., Pancratov, R., Rogatsky, I., Neubert, T.A., Fisher, E.A., and Garabedian, M.J. (2008). Phosphorylation of liver X receptor alpha selectively regulates target gene expression in macrophages. Mol. Cell. Biol. *28*, 2626–2636.

Uppal, H., Saini, S.P.S., Moschetta, A., Mu, Y., Zhou, J., Gong, H., Zhai, Y., Ren, S., Michalopoulos, G.K., Mangelsdorf, D.J., and Xie, W. (2007). Activation of LXRs prevents bile acid toxicity and cholestasis in female mice. Hepatology *45*, 422–432.

Wouters, K., van Bilsen, M., van Gorp, P.J., Bieghs, V., Lütjohann, D., Kerksiek, A., Staels, B., Hofker, M.H., and Shiri-Sverdlov, R. (2010). Intrahepatic cholesterol influences progression, inhibition and reversal of non-alcoholic steatohepatitis in hyperlipidemic mice. FEBS Lett. *584*, 1001–1005.

Wu, J.E., Basso, F., Shamburek, R.D., Amar, M.J.A., Vaisman, B., Szakacs, G., Joyce, C., Tansey, T., Freeman, L., Paigen, B.J., et al. (2004). Hepatic ABCG5 and ABCG8 overexpression increases hepatobiliary sterol transport but does not alter aortic atherosclerosis in transgenic mice. J. Biol. Chem. *279*, 22913–22925.

Wu, C., Hussein, M.A., Shrestha, E., Leone, S., Aiyegbo, M.S., Lambert, W.M., Pourcet, B., Cardozo, T., Gustafson, J.A., Fisher, E.A., et al. (2015). Modulation of macrophage gene expression via LXRα serine 198 phosphorylation. Mol. Cell. Biol. *35*, 2024–2034.

Xu, J., Xu, Y., Li, Y., Jadhav, K., You, M., Yin, L., Zhang, Y., Starr, S.P., Raines, D., Gao, B., et al. (2016). Carboxylesterase 1 is regulated by hepatocyte nuclear factor 4α and protects against alcohol- and MCD diet-induced liver injury. Sci. Rep. *6*, 24277.

Yee, J.K., Miyanohara, A., LaPorte, P., Bouic, K., Burns, J.C., and Friedmann, T. (1994). A general method for the generation of high-titer, pantropic retroviral vectors: Highly efficient infection of primary hepatocytes. Proc. Natl. Acad. Sci. USA *91*, 9564–9568.

Yuan, W., Xia, Y., Bell, C.G., Yet, I., Ferreira, T., Ward, K.J., Gao, F., Loomis, A.K., Hyde, C.L., Wu, H., et al. (2014). An integrated epigenomic analysis for type 2 diabetes susceptibility loci in monozygotic twins. Nat. Commun. *5*, 5719.

Zhao, B., Natarajan, R., and Ghosh, S. (2005). Human liver cholesteryl ester hydrolase: Cloning, molecular characterization, and role in cellular cholesterol homeostasis. Physiol. Genomics *23*, 304–310.

STAR★METHODS

KEY RESOURCES TABLE

REAGENT or RESOURCE	SOURCE	IDENTIFIER
Antibodies		
Mouse monoclonal anti-LXR alpha	Abcam	Cat#ab41902; Clone: PPZ0412; RRID: AB_776094
Rabbit polyclonal anti-LXRα/β	Laboratory of Knut R. Steffensen Pehkonen et al., 2012	N/A
Rabbit polyclonal anti-pSer196 LXRα	Torra et al., 2008	N/A
Rabbit polyclonal anti-HSP90α/β	Santa Cruz	Cat#sc-7947; RRID: AB_2121235
Rabbit monoclonal anti-LDLR [EP1553Y]	Abcam	Cat#ab52818; RRID: AB_881213
Mouse monoclonal anti-α-Tubulin	Sigma Aldrich	Cat#T5168; RRID: AB_477579
Goat anti-Rabbit Immunoglobulins/HRP	Dako	Cat#P0448; RRID: AB_2617138
Sheep anti-Mouse IgG - HRP	GE Healthcare	Cat#NA931; RRID: AB_772210
Rabbit monoclonal anti-TBLR1	Abcam	Cat#ab190796
Rabbit polyclonal anti-RXRα	Santa Cruz	Cat#sc-553; RRID: AB_2184874
Rabbit polyclonal anti-Pol II	Santa Cruz	Cat#sc-9001; RRID: AB_2268548
Rabbit polyclonal anti-RNA polymerase II CTD repeat YSPTSPS (phospho S2)	Abcam	Cat#ab5095; RRID: AB_304749
Rabbit polyclonal anti-Histone H3 (acetyl K27)	Abcam	Cat#ab4729; RRID: AB_2118291
Rabbit Immunoglobulin G	Sigma Aldrich	Cat#I5006; RRID: AB_1163659
Rat monoclonal anti-F4/80	Abcam	Cat#ab6640; RRID: AB_1140040
Biological Samples		
Healthy human liver tissue	University College London-RFH Biobank	REC reference 11/WA/0077
Chemicals, Peptides, and Recombinant Proteins		
T 0901317	Santa Cruz	CAS 293754-55-9
Supelco 37 Component FAME Mix	Sigma Aldrich	Cat#CRM47885
Bradford Reagent	Sigma Aldrich	Cat#B6916
T-PER Tissue Protein Extraction Reagent	Thermo Fisher Scientific	Cat#78510
DSG (disuccinimidyl glutarate)	Thermo Fisher Scientific	Cat#20593; CAS 79642-50-5
RNase A/T1	Thermo Fisher Scientific	Cat#EN0551
Proteinase K	Thermo Fisher Scientific	Cat#26160
RNAlater	Sigma Aldrich	Cat#R0901; CAS 7783-20-2
TRIzol Reagent	Thermo Fisher Scientific	Cat#15596026
Critical Commercial Assays		
High Fat-High Cholesterol diet	TestDiet Limited	Cat#58R7
Teklad chow diet (18% protein)	Harlan Laboratories	Cat#2018
LabAssay Cholesterol	Wako Diagnostics	Cat#294-65801
LabAssay Triglyceride	Wako Diagnostics	Cat#290-63701
Free Fatty Acid Assay Kit	Abcam	Cat#ab65341
Rat/Mouse Insulin ELISA	Millipore	Cat#EZRMI-13K
Mouse Total Bile Acids Assay Kit	Crystal Chem	Cat#80470
JumpStart Taq DNA Polymerase	Sigma Aldrich	Cat#D9307
PerfeCTa SYBR Green FastMix Low ROX	Quantabio	Cat#95071
qScript cDNA Synthesis Kit	Quantabio	Cat#95047
RT2 Profiler PCR Array Mouse Cytokines & Chemokines	QIAGEN	Cat#PAMM-150Z

(*Continued on next page*)

Continued

REAGENT or RESOURCE	SOURCE	IDENTIFIER
RT2 Profiler PCR Array Mouse Lipoprotein Signaling & Cholesterol Metabolism	QIAGEN	Cat#PAMM-080Z
Stranded mRNA-Seq Kit	Kapa Biosystems	Cat#KK8421
Crosslink IP Kit	Pierce	Cat#26147
Anti-FLAG M2 Magnetic Beads	Sigma Aldrich	Cat#M8823; RRID: AB_2637089
TUNEL Apoptosis Detection Kit - DAB	R&D Systems	Cat#4810-30-K
TBARS Assay Kit	Cayman Chemicals	Cat#10009055
ThruPLEX DNA-seq Kit	Takara Bio	Cat#R400523
QIAquick PCR Purification Kit	QIAGEN	Cat#28104
Deposited Data		
Chow livers RNA-Seq raw and analyzed data	This paper	GEO: GSE96650
HFHC livers RNA-Seq raw and analyzed data	This paper	GEO: GSE95359
ChIP-Seq data	This paper	GEO: GSE114104
Experimental Models: Cell Lines		
Human: HEK293T-VO,LXRα,S198A	Torra et al., 2008	N/A
Experimental Models: Organisms/Strains		
Mouse: WT and S196A	This paper	N/A
Oligonucleotides		
S196A genotyping primers: wild-type forward: GGTGTC CCCAAGGGTGTCCT, reverse: AAGCATGACCTGCACA CAAG, mutant forward: GGTGTCCCCAAGGGTGTCCG	This paper	N/A
Primers for qPCR, see Table S3	This paper	N/A
Primers for ChIP-qPCR analysis, see Table S4	This paper	N/A
ChIP-qPCR negative sequence primers	Active Motif	Cat#71011
Recombinant DNA		
Plasmid: LZRSpBMN-GFP, LZRSpBMN-GFP/LXRα, LZRSpBMN-GFP/S198A	Torra et al., 2008	N/A
Mouse vector: S196A$^{fl/fl}$	This paper	N/A
Software and Algorithms		
DESeq2	Anders and Huber, 2010	https://www.huber.embl.de/users/anders/DESeq/RRID: SCR_015687
Gene Set Enrichment Analysis (GSEA)	Subramanian et al., 2005	http://software.broadinstitute.org/gsea/datasets.jspRRID: SCR_003199
g:profiler	Reimand et al., 2016	https://biit.cs.ut.ee/gprofiler/
MultiExperiment Viewer (MeV)	Howe et al., 2011	mev.tm4.org/ RRID: SCR_001915
Heatmapper	Babicki et al., 2016	http://www2.heatmapper.ca/expression/
Venn Diagrams BEG tool	Laboratory of Yves Van de Peer	http://bioinformatics.psb.ugent.be/webtools/Venn/
ImageJ	National Institutes of Health	https://imagej.nih.gov/ij/ RID: SCR_003070
Eli (Easy Lipids) v1.0	This paper	http://www.ucl.ac.uk/muse/software
NHR-scan	Sandelin and Wasserman, 2005	http://www.cisreg.ca/cgi-bin/NHR-scan/nhr_scan.cgi
Integrative Genome Viewer	Robinson et al., 2011	https://www.broadinstitute.org/igv/ RRID:SCR_011793

CONTACT FOR REAGENT AND RESOURCE SHARING

Further information and requests for resources and reagents should be directed to and will be fulfilled by the Lead Contact, Inés Pineda-Torra (i.torra@ucl.ac.uk).

EXPERIMENTAL MODEL AND SUBJECT DETAILS

Generation of S196A transgenic animal models

The S196A floxed (S196A$^{fl/fl}$) mouse line was generated by Ozgene Pty Ltd (Bentley WA, Australia). The genomic sequence for the murine LXRα (Nr1h3) gene was obtained from the Ensembl Mouse Genome Server (http://www.ensembl.org//useast.ensembl.org/Mus_musculus/?redirectsrc=//www.ensembl.org%2FMus_musculus%2F), Ensembl gene ID: ENSMUSG00000002108. The mutant fragment, located on Exon 5, contains a serine-to-alanine mutation at Ser196 introduced by site-directed mutagenesis. The point mutant exon was delivered into an intronic site inside the targeting vector, placed in opposite orientation and thus without coding capacity (Figure S1A). The targeting construct was electroporated into the Bruce4 C57BL/6 ES cell line. Homologous recombinant ES cell clones were identified by Southern hybridization and injected into BALB/cJ blastocysts. Male chimeric mice were obtained and crossed to C57BL/6J females to establish heterozygous germline offsprings on a pure C57BL/6 background. The germline mice were crossed to a FLP Recombinase mouse line (Takeuchi et al., 2002) to remove the FRT flanked selectable marker cassette (Flp'd mice). Flp'd mice were then crossed with a transgenic C57BL/6 mouse strain carrying a Cre recombinase under the PGK-1 promoter (Koentgen et al., 2010), resulting in the inversion and insertion of the lox-flanked mutated (loxP) vector exon 5 region in the sense orientation, and deletion of the wild-type (WT) sequence in most adult cell lineages (S196A mice) while WT matching controls carry the WT sequence in the sense orientation (Figure S1D). Mice were genotyped by PCR analysis of ear biopsies (Figures S1D and S1E) using the Jumpstart Taq DNA Polymerase (Sigma Aldrich).

Animal husbandry

Animals were housed together in groups and maintained in a pathogen-free animal facility in a 12-h light-dark cycle in a temperature-controlled room (21.1 ± 1.1°C), with *ad libitum* access to water and food. Ten-week old female mice were used for all animal studies.

All procedures were carried under the UK's Home Office Animals (Scientific Procedures) Act 1986 and in accordance with the National Institutes of Health guidelines and the NYU Institutional Animal Care and Use Committee.

Culture of transfected HEK293T cells

HEK293T cells were obtained from the ATCC and maintained in Dulbecco's modified Eagle's medium with 10% Fetal Bovine Serum (FBS) and 20 μg/ml gentamicin. Recombinant retroviruses were produced by transfecting LZRSpBMN-GFP, LZRSpBMN-GFP/LXRα, or LZRSpBMN-GFP/S198A into 293GP cells (Yee et al., 1994). Cells infected with either the retroviral vector devoid of an LXRα sequence (VO [vector only]), the FLAG-tagged WT LXRα (LXRα), or phosphor mutant S198A (S198A) were sorted for green fluorescent protein expression by fluorescence-activated cell sorting. These cell lines are from human female origin and represented a pool of multiple LXRα-expressing clones.

Human liver tissue

Frozen liver biopsies from adult males with colon carcinoma undergoing lobectomies were obtained from the UCL-RFH Biobank (approved by UCL–Royal Free Hospital BioBank Ethical Review Committee, 11/WA/0077). Study was approved by the local ethical board (NRES Rec Reference NC2015.020.). Each participant gave written informed consent. Storage of samples complied with the requirements of the Data Protection Act of 1998 and the Human Tissue Act of 2004.

METHOD DETAILS

Diet and drug studies and tissue collection

Ten-week old WT and S196A female mice were fed *ad libitum* a High Fat-High Cholesterol (HFHC) diet (17,2% Cocoa Butter, 2,8% Soybean Oil, 1,25% Cholesterol, 0,5% Sodium Cholate; AIN-76A/Clinton Diet #4, Test Diet Limited, UK) or a chow diet (18% Protein, 6.2% Fat, 0% Cholesterol; Harlan Laboratories) for 6 or 12 weeks. For ligand activation studies, WT and S196A female mice were administered by oral gavage 50 mg/kg/day of T0901317 (Santa Cruz Biotechnology) in 0.5% methylcellulose or vehicle alone for four days.

Mice were fasted overnight prior to sacrifice. Blood was collected by cardiac puncture and plasma was aliquoted and frozen at -80°C. Tissue was dissected, weighted and frozen at - 80°C or placed in RNAlater (Sigma Aldrich).

Plasma and liver lipids

Frozen livers (50 mg) were homogenized in 250 mM sucrose, 2 mM EDTA, 10 mM Tris buffer using ceramic beads in a Minilys Tissue Homogenizer (Bertin Corp.). Triglycerides and Cholesterol were extracted with Isopropanol or Chloroform:Methanol (1:1) solutions, respectively. Non Esterified Free Fatty Acids (NEFAs) were extracted by incubating liver homogenates with 1% Triton-100X and chloroform solution. Plasma and hepatic total cholesterol, triglyceride levels (Wako Diagnostics), and NEFAs (Abcam) were determined by colorimetric enzymatic assay kits as per the manufacturer's recommendations. Hepatic lipid content was normalized to protein concentration, quantified using the Bradford Protein Assay. To this end, liver homogenates were diluted in water and incubated with Bradford Reagent (Sigma Aldrich) for 30 minutes at room temperature. Absorbance was measured at 595 nm in Microplate Reader.

Plasma glucose and insulin

Blood glucose measurements (Accu-Chek, Roche Diagnostics) were taken from tail blood samples after overnight fasting. Plasma insulin concentration was measured using a rat/mouse Insulin Enzyme-Linked ImmunoSorbent Assay (ELISA) kit (Millipore), which contained a 96-well plate pre-coated with a pre-titered amount of monoclonal mouse anti-rat insulin antibodies. Quantification of immobilized antibody-enzyme conjugates was performed by monitoring horseradish peroxidase activities in the presence of the substrate 3,3′,5,5′-tetramethylbenzidine; which was measured spectrophotometrically by the increased absorbency at 450 nm, corrected from the absorbency at 590 nm. Amount of captured insulin was derived by interpolation from a reference curve generated in the same assay with reference standards of known concentrations of rat insulin (Millipore).

Hepatic fatty acid profiles

Liver fatty acid content from WT and S196A mice fed a HFHC diet were assayed by gas liquid chromatography with flame ionization detection by AS Vitas (Oslo, Norway). Internal standard triheptadecanoin was added and fatty acids were methylated into methyl esters (FAMEs) with MeOH HCl and extracted with hexane. Analyses were performed on an Agilent 7890A GC and a 7683B automatic liquid sampler and flame ionization detection (Agilent Technologies, USA). Separations were obtained using a SP-2380 column. Fatty acid content was calculated based on the area percentage of peaks and response factors relative to 18:0. An external standard containing known amounts of relevant FAMEs (Supelco 37 component FAME Mix) was included in each run to correct for differences in fatty acid response factors. Results were normalized to protein content. The ratio of 16:0 to 18:2 n-6 was used to calculate a *de novo* lipogenesis index (Chong et al., 2008). The total saturated fatty acid content was calculated as the sum of 12:0, 14:0, 15:0, 16:0, 17:0, 18:0, 20:0 and 22:0. The total unsaturated fatty acid content was calculated as the sum of ω9 (16:1 c9, 18:1 c9, 20:1 n-9), ω6 (18:2 n-6, 18:3 n-6, 20:2 n-6, 20:3 n-6, 20:4 n-6) and ω3 (18:3 n-3, 20:5 n-3, 22:5 n-3, 22:6 n-3) fatty acids.

Hepatic bile acid quantification

50 mg of frozen livers were homogenized in 75% ethyl alcohol (VWR) using a Dounce homogenizer and homogenates were incubated at 50°C for 2 hours. Tubes were then centrifuged for 10 min. at 6000 x g, 4°C and supernatant was transferred onto a clean tube. Total bile acid concentration in supernatants was quantified using the Mouse Total Bile Acids kit (Crystal Chem.), as per manufacturer's instructions. Hepatic bile acids were normalized to total protein concentration, quantified using the Bradford Protein Assay.

Oxysterol LC-MS analysis

Protein was precipitated from plasma with 480 pM of 24R/S hydroxycholesterol-d7 (24R/S-d7), 25-hydroxycholesterol-d6 (25-d6), 27-hydroxycholesterol-d6 (27-d6), 22R-hydroxycholesterol-d7 (22R-d7) and 1214 pM 24-25-epoxycholesterol-d6 (2425e-d6) as internal standards (Avanti Polar Lipids, Alabaster, AL, USA). Sample clean-up was conducted offline, using solid phase extraction (SPE, SilactSPE C18 100 mg, Teknolab, Ski, Norway) and dried at 30°C, re-dissolved in 2-propanol and derived (Roberg-Larsen et al., 2014). Samples and calibration solutions were analyzed using an Ultimate 3000 UHPLC connected to an Advantage QqQ (both Thermo Fisher, Waltham, MA, USA) equipped with an Automatic filtration and filter back-flush SPE add-on (Roberg-Larsen et al., 2017). Injection volume was 100 μL and oxysterols were retained on-line on a Hotsep Kromasil C18 100 Å 1 mm ID x 5 mm SPE. Loading mobile phase was 0.1% formic acid (FA, Sigma Aldrich, St. Louis, MI, USA) in type 1 water with a flow rate of 100 μL/min. Loading time was 2 minutes to remove excess derivatization reagent. The valve was automatically controlled by Chromeleon software. Separation of the oxysterols was achieved on an ACE 3 C18 1 mm ID x 100 mm column using a gradient. Mobile phase A and B was 0.1% FA in type 1 water and 0.1% FA in MeOH, respectively and flow rate was 75 μL/min. Gradient started at 70% B and increased to 80% B in 10 minutes, followed by an increase to 95% B in 1 minute and was hold at 95% for 10 minutes. Column temperature was 30°C. Total analysis time per sample (including injection and column reconditioning) was 27 minutes.

Faecal cholesterol quantification

Dried faeces were weighed (40 mg) and grounded with a mortar and pestle. Powdered faeces were then resuspended in Phosphate Buffer Saline and solution was mixed with 5 mL of a Chloroform:Methanol (1:1) solution (VWR). Tubes were left to incubate for 10 minutes at room temperature while shaking, and were then centrifuged (3000 x g, 10 min, room temperature). Supernatants were transferred onto fresh tubes and were allowed to evaporate to dryness by placing the open tube at 65°C. Dried cholesterol remnants were then resuspended with 200 μL of isopranol + 10% Triton-100X solution (Fisher Bioreagents). Total cholesterol levels were determined using a colorimetric kit (Wako Chemicals).

Gene expression analysis

Total RNA from was extracted with TRIzol Reagent (Invitrogen). Sample concentration and purity was determined using a NanoDrop 1000 Spectrophotometer and cDNA was synthesized using the qScript cDNA Synthesis Kit (Quanta). Specific genes were amplified and quantified by quantitative PCR (qPCR), using the PerfeCTa SYBR Green FastMix (Quanta) on an MX3000p system (Agilent). Primer sequences are available on Supplemental table S3. The relative amount of mRNAs was calculated using the comparative Ct method and normalized to the expression of cyclophilin (Pourcet et al., 2016). Mouse Cytokines & Chemokines and Lipoprotein Signaling & Cholesterol Metabolism RT2 Profiler PCR Arrays were performed per the manufacturer's instructions (QIAGEN). Briefly,

cDNA was synthesized using an RT^2 HT first strand kit (QIAGEN), and qPCR analysis was performed using RT2 SYBR Green ROX qPCR Mastermix (QIAGEN). The relative amount of mRNAs was calculated using the comparative Ct method and normalized to an average of five housekeeping genes.

RNA sequencing studies

Total RNA was extracted using TRIzol (Life technologies) and cDNA libraries were prepared using the Stranded mRNA-Seq Kit (Kapa Biosystems). Briefly, poly-A tailed RNA was purified using paramagnetic oligo-dT beads from 200 nanograms of total RNA, with a RNA Integrity Number above 7.5 as determined by the Agilent Bioanalyzer. The purified RNA was chemically fragmented and cDNA was synthesized using random primers (Kapa Biosystems). Adaptor-ligated DNA library was amplified with 12 cycles of PCR and library fragment was estimated using the Agilent TapeStation 2200.Library concentration was determined using the Qubit DNA HS assay (Life Technologies). Libraries were sequenced on an Illumina NextSeq 500, NCS v2.1.2 (Illumina) with a 43bp paired end protocol. Basecalling was done using standard Illumina parameters (RTA 2.4.11). Sequencing and pipeline analysis was performed by UCL Genomics (London, UK). Reads were demulitplexed using Illumina's bcl2fastq v2.17 and aligned using STAR v2.5.0b to the mouse GRCm38/mm10 reference sequence. Transcript abundance was estimated using Illumina's RnaReadCounter tool and differential expression analysis performed with DESeq2, which uses the Benjamin-Hochberg method for multiple testing correction (Anders and Huber, 2010). Pathway enrichment analysis was performed with the Gene Set Enrichment Analysis (GSEA) software's pre-ranked module (Subramanian et al., 2005) and g:profiler (Reimand et al., 2016). Top regulated genes were confirmed by qPCR on a separate set of liver samples from HFHC-fed mice. Heatmaps were created using raw gene count values with the MultiExperiment Viewer (MeV) software (Howe et al., 2011). Clustered heatmaps of normalized gene counts were created with Heatmapper Expression tool (Babicki et al., 2016) and Venn diagrams using a BGE tool.

Western Blotting

Whole liver samples were homogenized with T-PER lysis buffer (78510, Thermo Fisher Scientific) supplemented with protease and phosphatase inhibitors. Total cellular protein lysates (30μg) were loaded onto a 10% SDS-PAGE gel, electrophoresed and transferred onto a PVDF membrane. For immunoprecipitations studies, single cell suspensions from livers were immunoprecipitated with antibodies that specifically recognize human (LXRα, ab41902 Abcam) or murine (LXRα/β) (Pehkonen et al., 2012) receptors previously crosslinked to a column with Protein A/G Agarose following the manufacturer's protocol (Pierce). Phospho-Ser196 specific rabbit polyclonal antibody (Torra et al., 2008), mouse α-LXRα monoclonal antibody (ab41902, Abcam), α-Hsp90 polyclonal (sc-7947, Santa Cruz), α-LDLR monoclonal (Abcam, ab52818) and anti-α-Tubulin monoclonal antibody (Sigma Aldrich, T5168) were used for immunoblotting. Anti-rabbit (PO448, Dako) or anti-mouse (NA931VS, GE Healthcare) horseradish-peroxidase-tagged antibodies were used for secondary binding and chemiluminescence (ECL 2 Western Blotting Substrate, Pierce) was used to visualize proteins.

For co-immunoprecipitation studies, HEK293T-LXRα, HEK-S198A or HEK293T-Vo cells expressing FLAG-tagged receptors as in (Torra et al., 2008) were lysed and crude nuclear pellets were obtained. Supernatants containing nuclear proteins were incubated with FLAG antibody-conjugated agarose beads (Sigma Aldrich). Bead-associated proteins associated were eluted in TBS and immunoblotted with α-TBLR1 (ab190796, Abcam) or α-LXRα (ab41902, Abcam) antibodies.

Histopathological analysis

Formalin-fixed, paraffin-embedded mouse livers were cut and stained with hematoxylin and eosin (H&E) or Picrosirius Red (Abcam) dyes. Liver histology was blindly scored by an independent histopathologist based on three semiquantitative items: steatosis (0–3), lobular inflammation (0–3) and hepatocellular ballooning (0–2) (not shown) (Liang et al., 2014). Stained sections were scanned with NanoZoomer Digital slide scanner (Hamamatsu) and quantification of Picrosirius red-stained areas was performed using ImageJ on three independent areas per section. Data is represented as the average positively-stained percent of area of interest.

Hepatic macrophage content was assessed by quantification of F4/80 positively-stained areas (ab6640, Abcam).

Lipid droplet identification

Identification and quantification of lipid droplets were made with the help of Eli (Easy Lipids) v1.0, an in-house software developed between the Multiscale Cardiovascular Engineering (MUSE) and Dr Pineda-Torra's groups at UCL. This software uses a method based on the Hough Transform (Duda and Hart, 1972) for the identification of the droplets estimating the centers and radii of each of them. A final report is generated with the dimensions of the droplets (i.e., diameter and area) including a histogram describing the frequency of lipid vacuoles within specified diameter ranges.

TUNEL staining

Apoptosis was detected *in situ* using a terminal deoxynucleotidyl transferase dUTP nick end-labeling (TUNEL) assay as per the manufacturer's instructions (R&D Systems). Paraffin-embedded liver tissue sections were incubated with a specific TdT enzyme that incorporates biotin on exposed nucleotides after DNA fragmentation. Biotin labeling was later achieved using Streptavidin-Fluorescein and sections were imaged using the Axio Imager.A1 Digital Microscope (Zeiss). Four different areas per slide were photographed at a magnification of 200X and intensity of staining was quantified by ImageJ.

Lipid peroxidation quantification

Thiobarbituric Acid Reactive Substances (TBARS) were measured in about 25 mg of frozen liver as per manufacturer's instructions (Cayman Chemicals). Briefly, lipid peroxidation was quantified by the reaction of Malondialdehyde (MDA), a product of lipid peroxidation, with thiobarbituric acid (TBA) to form a colorimetric (532 nm) product, proportional to the MDA present in the sample. Levels of MDA were normalized to total protein levels, quantified by the Bradford Assay.

LXRα proteomic analysis

HEK293T cells expressing vector only (Vo), FLAG-hLXRα or FLAG-hLXRα-S198A (Shrestha et al., 2016) were treated with 1 μM T0901317 for 8 hours. Cells were lysed in hypotonic buffer (10 mM HEPES, 1.5 mM MgCl2, 10 mM KCl, pH 7.9) and FLAG-LXRα was immunoprecipitated from the nuclear extracts using agarose beads conjugated to FLAG antibody (Sigma). The beads were then incubated in 50 μL of TBS with 0.5 mg/ml FLAG peptide (Sigma F3165) and the proteins in the supernatant were precipitated with TCA overnight. The TCA precipitate was processed and then subjected to analysis by Multidimensional Protein Identification Technology and LTQ and LTQ orbitrap mass spectrometry (Shrestha et al., 2016).

Screening of potential LXREs

Screening for potential DR4 sequences was performed using the available web-based software NHR Scan (http://www.cisreg.ca/cgi-bin/NHR-scan/nhr_scan.cgi) (Sandelin and Wasserman, 2005), which predicts potential nuclear hormone receptor binding sites on a given genomic sequence. Input sequences (gene body or sequences 30 kb upstream of transcription start site) were obtained from UCSC Genome Browser database and submitted under FASTA format. Plausible DR4 sequences were then chosen based on similarity to a published consensus sequence for the murine LXRE (Boergesen et al., 2012).

ChIP-qPCR and ChIP-sequencing

Fresh mouse livers (n = 3/genotype) were crosslinked with 2 mM disuccinimidyl glutarate (DSG) for 30 min, followed by 1% formaldehyde for 10 min at room temperature. The reaction was stopped with glycine at a final concentration of 0.125 M for 5 min. Single cell suspension were obtained by grinding liver pieces through a 70 μM cell strainer, and nuclei were isolated by incubating cell preparations for 10 minutes at 4°C with the following lysis buffers: Buffer 1 (50 mM HEPES-KOH, pH 7.5, 140 mM NaCl, 1 mM EDTA, 10% glycerol, 0.5% NP-40 and 0.25% Triton X-100), Buffer 2 (10 mM Tris-HCl, pH 8.0, 200 mM NaCl, 1 mM EDTA and 0.5 mM EGTA), and Buffer 3 (10 mM Tris-HCl, pH 8.0, 100 mM NaCl, 1 mM EDTA, 0.5 mM EGTA, 0.1% Na-deoxycholate, and 0.5% N-Lauroylsarcosine). Pellets resuspended in 300 μL of lysis buffer 3 were sonicated for 40 cycles (30 s ON/OFF) in the UCD-300 Bioruptor (Diagenode), to generate DNA-fragment sizes of 0.2–0.5 kb. The following antibodies were used for immunoprecipitations: RXRα (sc-553, Santa Cruz), Pol II (sc-9001, Santa Cruz), Pol II-S2P (ab5095, Abcam), pS196-LXRα (Torra et al., 2008) LXR (Pehkonen et al., 2012) and control rabbit IgG (I5006, Sigma Aldrich). Following RNase A (Thermo Fisher Scientific) and proteinase K (Thermo Fisher Scientific) treatment, immunoprecipitated DNA was purified using the QIAquick PCR purification kit (QIAGEN) and analyzed by quantitative real-time PCR (primer sequences are listed in Table S3) and relative occupancies were normalized to input DNA (fold difference = $2^{-\text{Ct-sample-Ct-input}}$). To control for non-specific binding, a 82 base pair fragment in a gene desert in chromosome 6 (ActiveMotif) was used. Triplicate samples for ChIP-seq were sonicated in Diagenode Pico sonicator and immunoprecipitated with H3K27Ac (ab4729, Abcam). For library preparation, 2 ng of immunoprecipitated DNA was processed using ThruPLEX® DNA-seq Kit (Rubicon) according to manufacturer's protocols, and 50SE reads were obtained in the Illumina HiSeq 2000 (Illumina). Sequencing files (fastq files), provided by the Bioinformatics and Expression Analysis (BEA) core facility (Karolinska Institutet), and raw data from published ChIP-seq data (GSE35262) were aligned to the NCBI37/mm9 version of the mouse reference genome, using Bowtie (Fan et al., 2016). The sequencing tags were then read and imported to the HOMER (Hypergeometric Optimization of Motif EnRichment, http://homer.ucsd.edu/homer) package. Peaks were identified using HOMER with default settings, and peak overlap was calculated by merging all individual peak files for every experiment. and peak heights were normalized to the total number of uniquely mapped reads and displayed in Integrative Genomics Viewer (Robinson et al., 2011) as the number of tags per 10 million tags.

QUANTIFICATION AND STATISTICAL ANALYSIS

Statistical analysis was performed using Prism (GraphPad) and Microsoft Excel. Unless otherwise states, data is presented as mean ± SEM. For multiple comparisons, significance was assessed by single variance ANOVA followed by Student's t test. The F-statistic (dfbetween = 3, dfwithin = 15) and the P value for the significant main effect are shown. Differences were considered significant at $p < 0.05$ by a two-tailed Student t test. For distribution of liver lipid droplets, areas were compared by chi-square for trend.

Statistical details, significance and n values can be found in the figure legends.

DATA AND SOFTWARE AVAILABILITY

The accession numbers for gene expression RNaseq (chow and HFHC diet) and H3K27Ac ChIPSeq data reported in this paper are GEO: GSE96650 (chow), GEO: GSE95359 (HFHC), and GEO: GSE114104 (ChIPSeq).

A trial of Eli v1.0 is currently available upon request on the MUSE website at UCL (http://www.ucl.ac.uk/muse/software).

Cell Reports, Volume *26*

Supplemental Information

Impaired LXRα Phosphorylation Attenuates Progression of Fatty Liver Disease

Natalia Becares, Matthew C. Gage, Maud Voisin, Elina Shrestha, Lucia Martin-Gutierrez, Ning Liang, Rikah Louie, Benoit Pourcet, Oscar M. Pello, Tu Vinh Luong, Saioa Goñi, Cesar Pichardo-Almarza, Hanne Røberg-Larsen, Vanessa Diaz-Zuccarini, Knut R. Steffensen, Alastair O'Brien, Michael J. Garabedian, Krista Rombouts, Eckardt Treuter, and Inés Pineda-Torra

Figure S1. Related to Figures 1 & 2

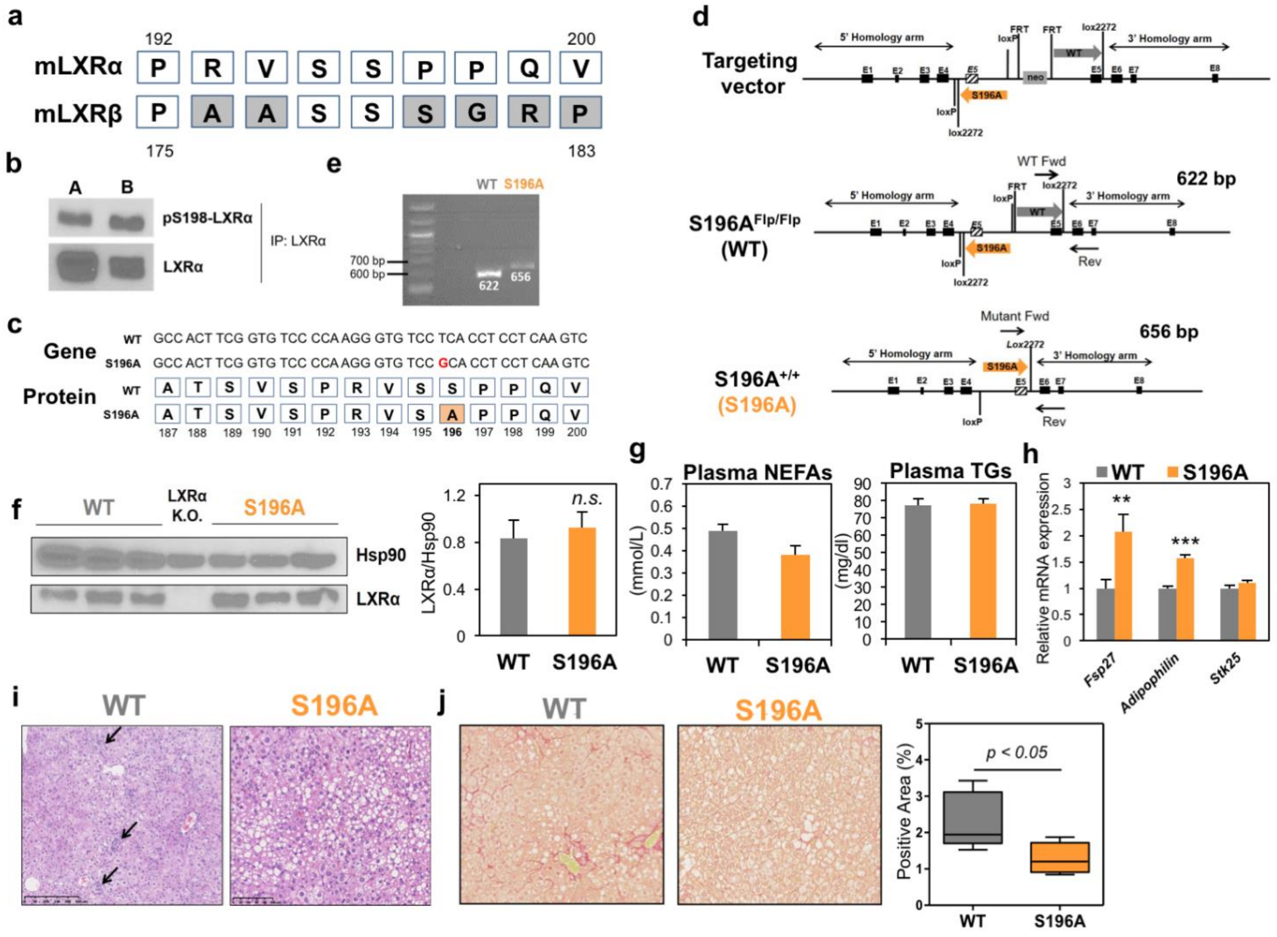

(a) Alignment of the murine LXRα and LXRβ showing differences in S196 phosphorylation motifs. **(b)** LXRα phosphorylation at Ser198 and total LXRα levels in human liver lysates (n=2) by immunoblotting. **(c)** WT and S196A genomic and protein sequence alignment of the murine LXRα depicting the single-site mutation at S196A. **(d)** Targeting construct containing the loxP and FRT sites, the predicted homologous recombinant alleles and the resulting WT and LXRα knock-in locus incorporating the mutated sequence. Diagram also shows oligos used for genotyping and product size. **(e)** Gel electrophoresis of DNA amplified products using the corresponding primers.

(f) Total LXRα and Hsp90 detected by immunoblotting in WT, S196A and LXRα knock-out livers. Densitometry quantification on LXRα levels normalised to Hsp90 loading control (n=3). (**g**) Plasma non-esterified fatty acids (NEFAs) and triglycerides (TGs) levels from WT and S196A mice on HFHC diet (n=5-6). Data are means ± SEM. **(h)** Hepatic gene expression of lipid droplet proteins from WT or S196A mice (n=6). Results shown normalized to cyclophilin and relative to WT set as 1. Data represents means ± SEM. * $p < 0.05$ or ** $p < 0.005$ relative to WT determined by Student's t-test. (**i**) Representative image of H&E-stained liver of WT and S196A mice fed a HFHC diet for 6 weeks. Arrows are pointing several inflammatory loci in WT liver. Scale bar at 50 μM. **(j)** Representative images of Picrosirius Red-stained liver sections from WT and S196A mice on a HFHC diet for 12 weeks. Images are at 200x magnifications. Quantification of Picrosirius red-stained areas on three independent areas per section (n=6). Data represent means ± SEM.

Figure S2. Related to Figures 2 & 3.

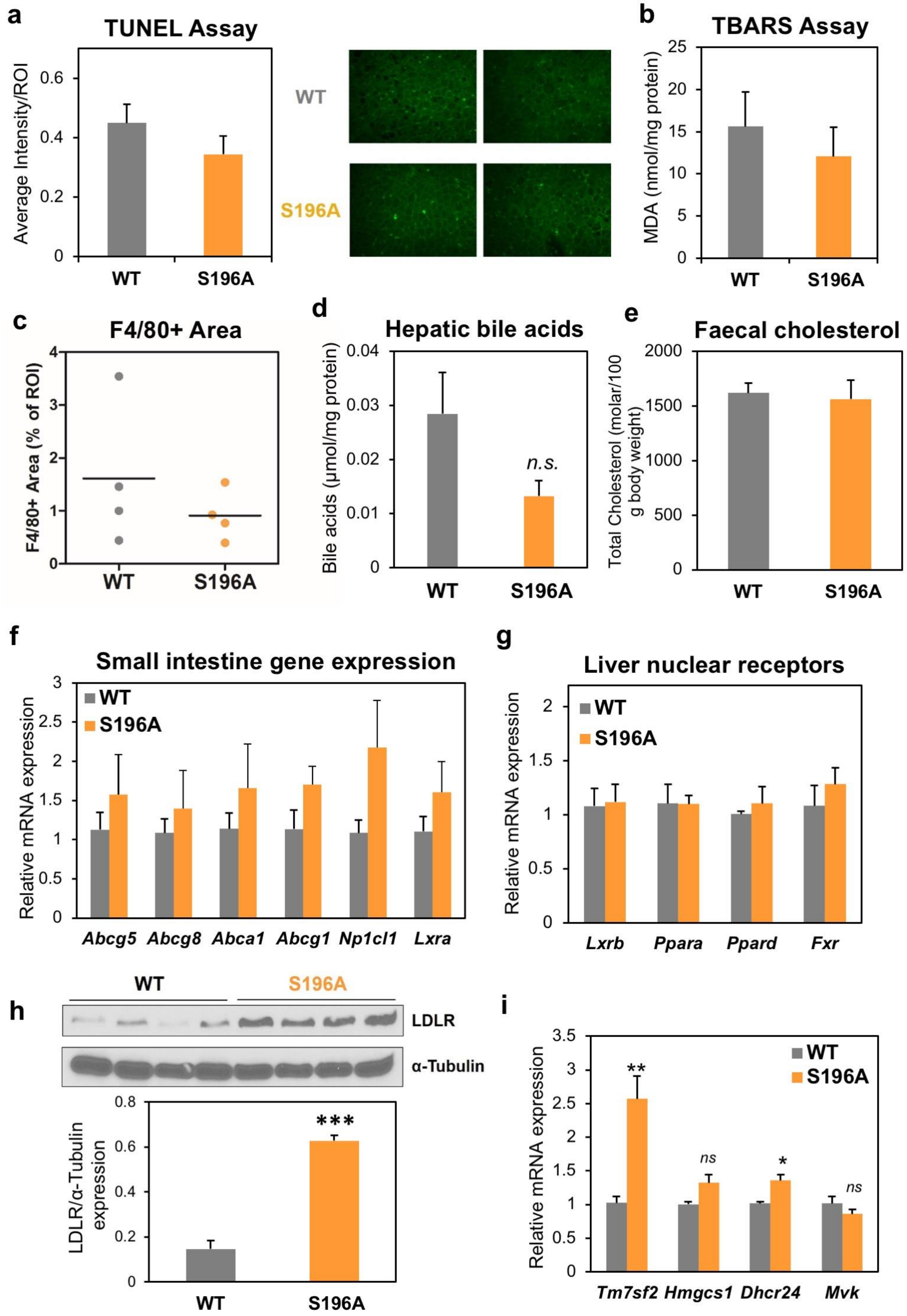

(a) Hepatic cell apoptosis assessed *in situ* by Direct DNA Fragmentation (TUNEL) Assay (n=6/group) *(Right)*. Representative images of TUNEL-stained liver sections from WT and S196A mice at 200x magnification (*Left).* **(b)** Hepatic lipid peroxidation shown as MDA levels in WT and S196A livers (n=6) normalised to protein levels in tissue homogenates. **(c)** Quantification of F4/80-positively stained areas in liver sections of WT and S196A mice (n=4) at 200x magnification. Dots represent average of three independent areas per animal. (**d**) Hepatic bile acid levels from WT and S196A mice fed a HFHC diet for 6 weeks (n=6). Values normalized to protein levels in liver homogenates. **(e)** Total cholesterol levels of faeces from WT and S196A mice (n=4). Values are shown per 100 g of dried faeces and normalized to animal body weight. **(f)** Small intestine and **(g)** **(i)** hepatic gene expression from WT or S196A mice fed a HFHC diet for 6 weeks (n=6). Results shown normalized to cyclophilin levels and relative to WT. **(h)** LDL-Receptor (LDLR) and α-Tubulin levels detected by immunoblotting in WT and S196A livers. Densitometry was performed on the levels of LDLR and normalised to the levels of the housekeeping α-Tubulin (n=4). Data represent means ± SEM. * $p < 0.05$ or ** $p < 0.005$ relative to WT determined by Student's t-test.

Figure S3. Related to Figure 4.

a

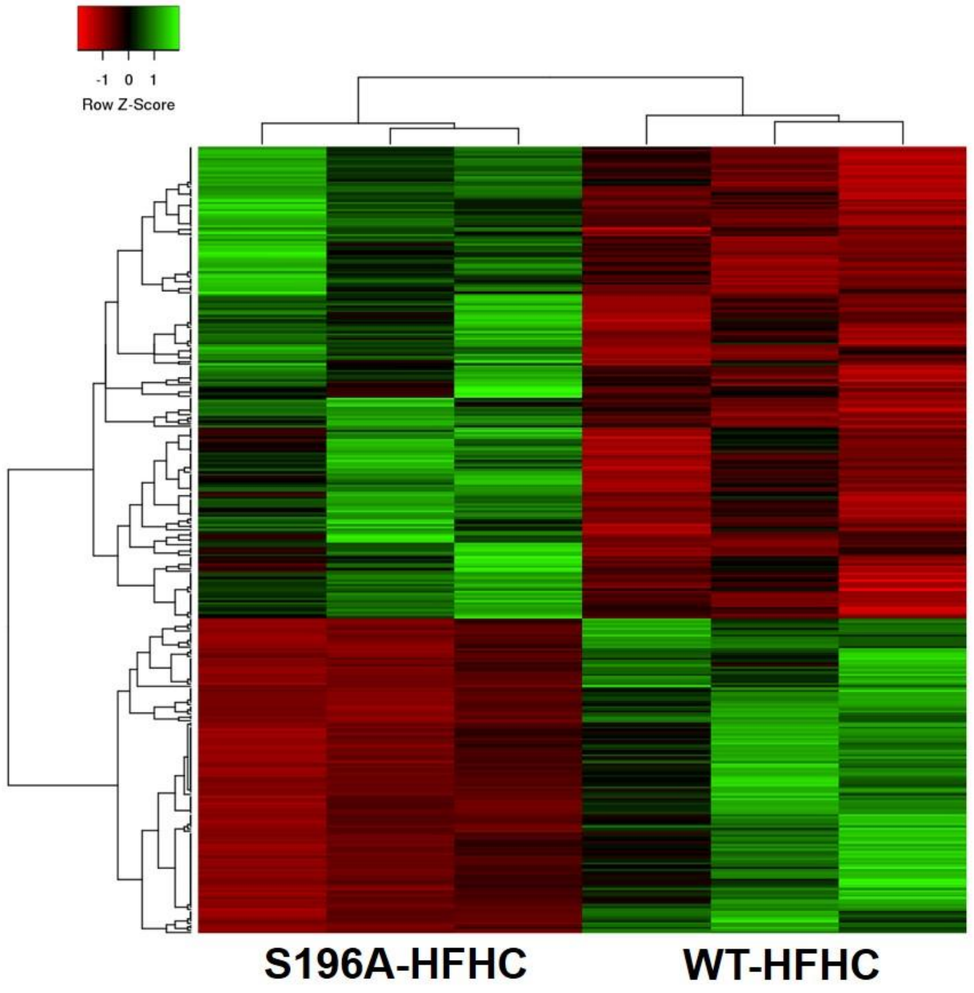

b

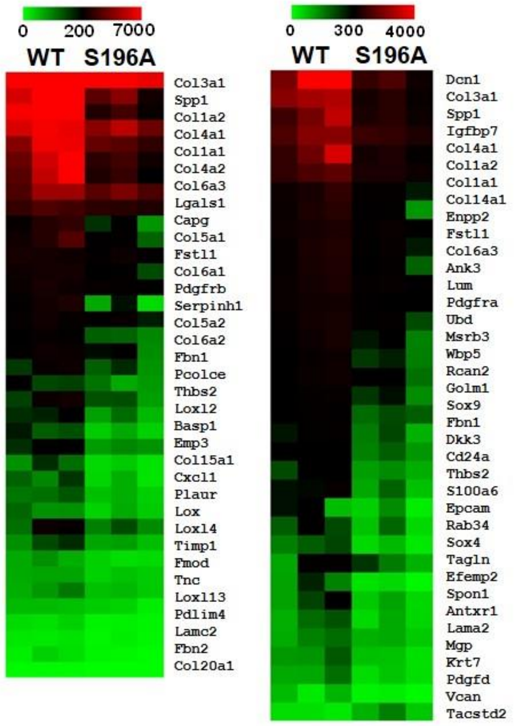

(a) Clustered heatmap of hepatic RNAseq normalised gene counts in WT and S196A mutant mice (n=3/genotype) of regulated genes in response to a HFHC diet. **(b)** Heatmaps of hepatic RNAseq normalised gene counts (n=3/genotype) for fibrosis (left) and mouse hepatic expression of genes previously identified to be part of a signature that distinguishes human NAFLD (right).

Figure S4. Related to Figure 4.

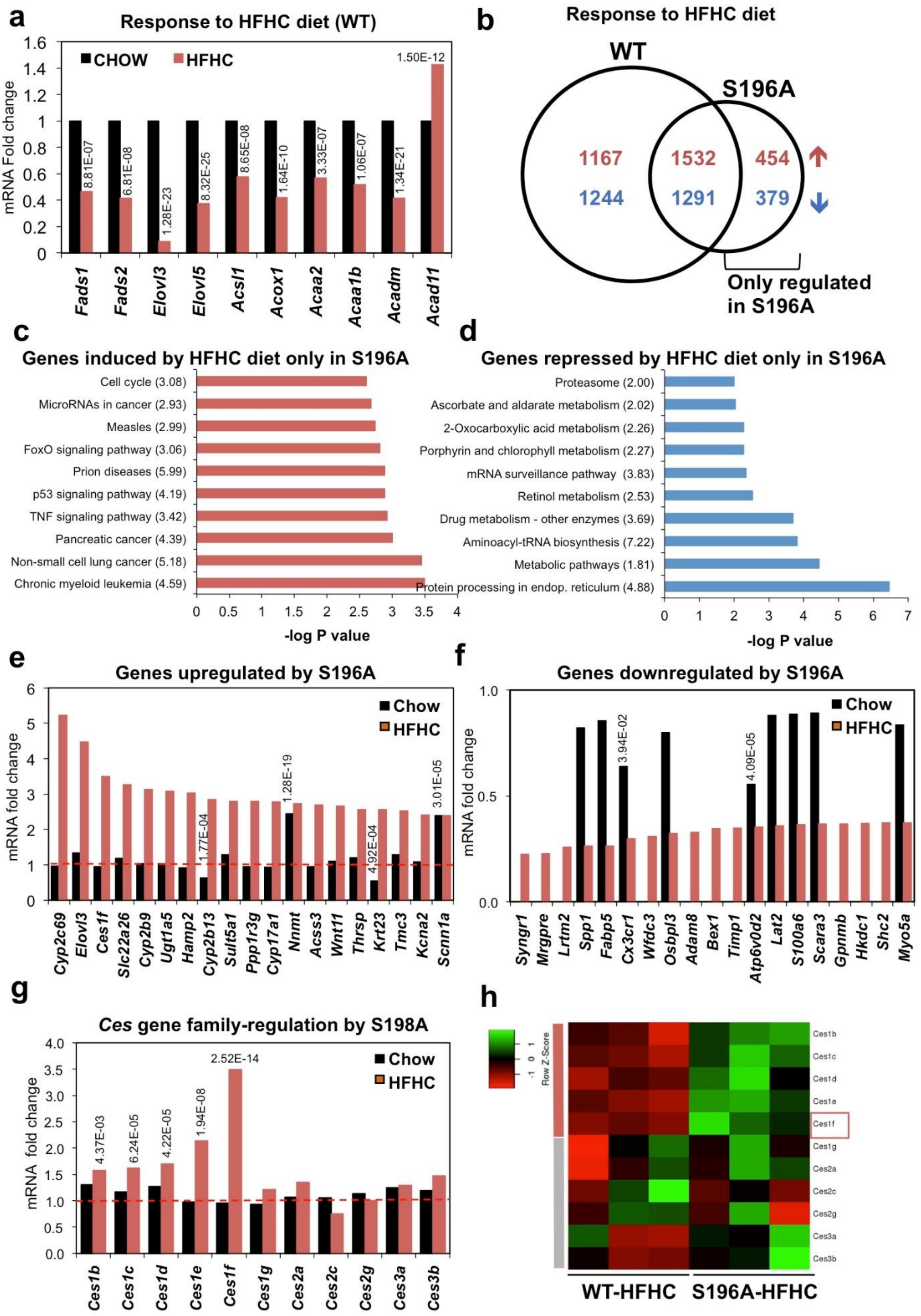

(a) Fold change of hepatic RNAseq gene counts of fatty acid genes in response to the HFHC diet compared to chow (set as 1) in WT mice (n=3/group). Adjusted *p* values (FDR 0.05) are shown. **(b)** Venn diagram of genes induced (red) or reduced (blue) in response to HFHC diet in WT or S196A mice. Significance set at $p \leq 0.05$. **(b,c)** Pathway analysis considering group of genes induced **(b)** or repressed **(c)** by HFHC diet only in S196A mice. Bar graphs shows top ten KEGG Pathways with enrichment score between brackets. **(d)** Fold change of hepatic RNAseq normalised gene counts of top upregulated genes in S196A compared to WT mice (from Fig. 4) on chow or HFHC diet (n=3/genotype). Shown are p values of genes differentially expressed between WT and S196A mice on chow. All genes shown are significantly regulated on the HFHC diet ($p<0.05$). Red bar set at fold change=1 indicates no change in gene expression between WT and S196A mice. **(e)** Fold change of hepatic RNAseq normalised gene counts of top downregulated genes in S196A compared to WT mice (from Fig. 4) fed chow or HFHC diet (n=3/genotype). For gene expression on chow, p values of genes differentially expressed between WT and S196A are shown. Data is not shown for those genes minimally expressed in chow. For gene expression on the HFHC diet, all genes depicted are significantly reduced ($p<0.05$). **(g)** Fold change of hepatic RNAseq normalised gene counts for Ces gene family members comparing WT and S1986A genotypes by diet (n=3/group). Shown are p values of genes differentially expressed on a HFHC diet. Red bar set at fold change=1 indicates no change in gene expression between WT and S196A mice. **(h)** Heatmap of hepatic RNAseq normalised gene counts in WT and S196A mutant mice (n=3/genotype) of Ces family genes on a HFHC diet. Red and grey bars indicate positively regulated or unchanged genes, respectively. Highest regulated Ces member (Ces1f) is shown boxed.

Figure S5. Related to Figure 5.

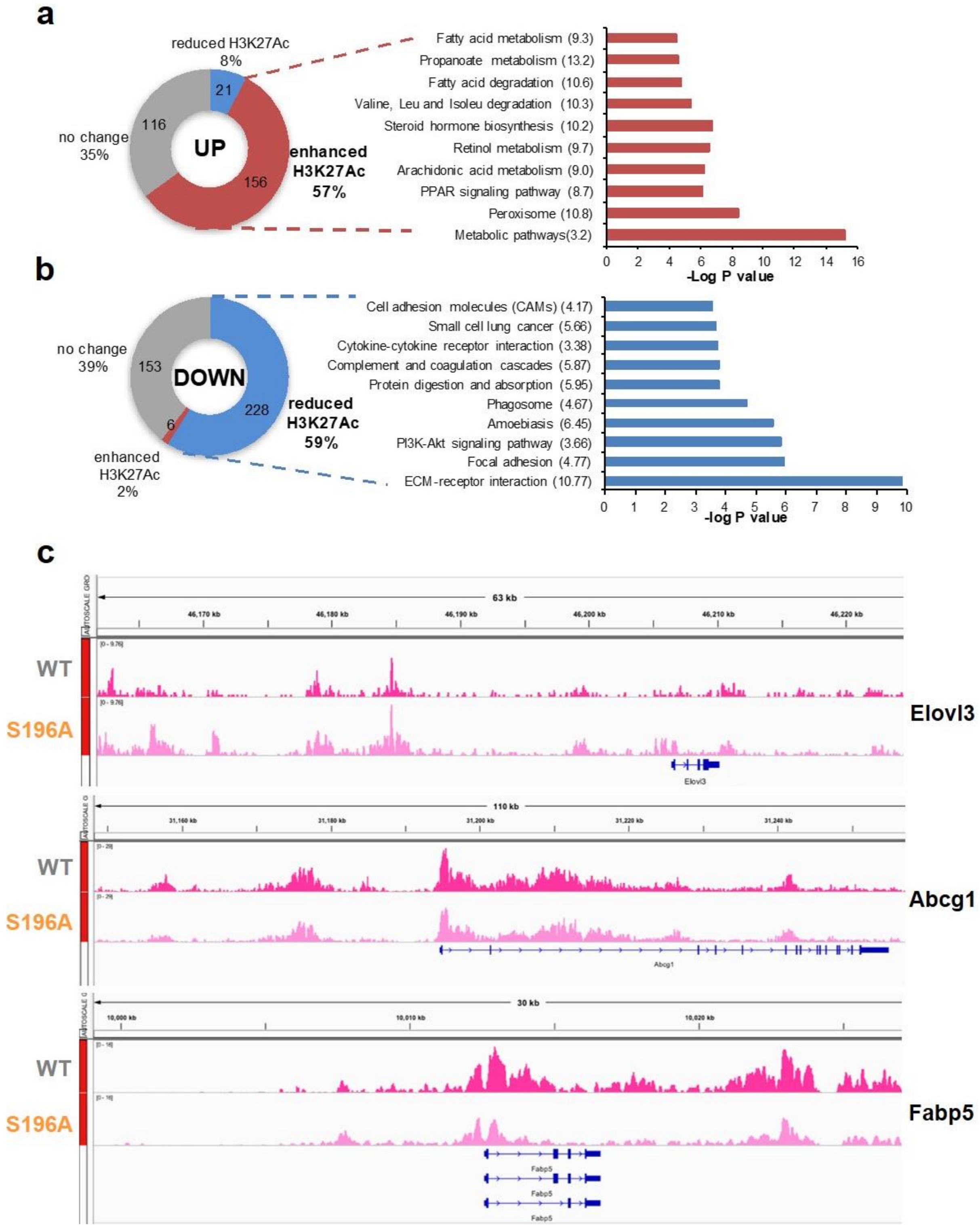

(a-b) Proportion of genes that are up-(a) or down- (b) regulated in S196A vs WT fatty livers showing changes in H3K27Ac by ChIPseq analysis. Number of genes are shown inside the graph. Bar graphs shows top ten KEGG Pathways (FDR<0.05) with enrichment scores between brackets. **(c)** Representative H3K27Ac ChIP-seq read alignment tracks in WT and S196A HFHC-fed livers for up-regulated (*Elovl3*) and down-regulated (*Abcg1* and *Fabp5*) genes in S196A livers.

Figure S6. Related to Figure 6.

a

VO	LXRα	S198A	Peptide
0	13	37	NCoR
0	4	22	TBLR1

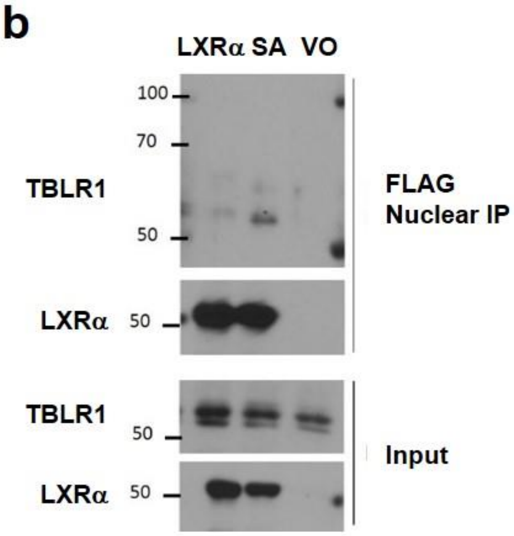

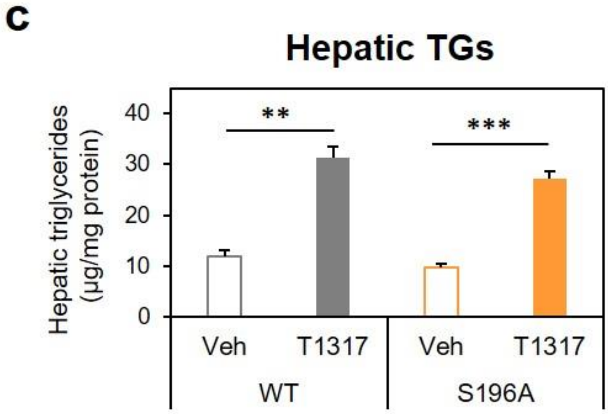

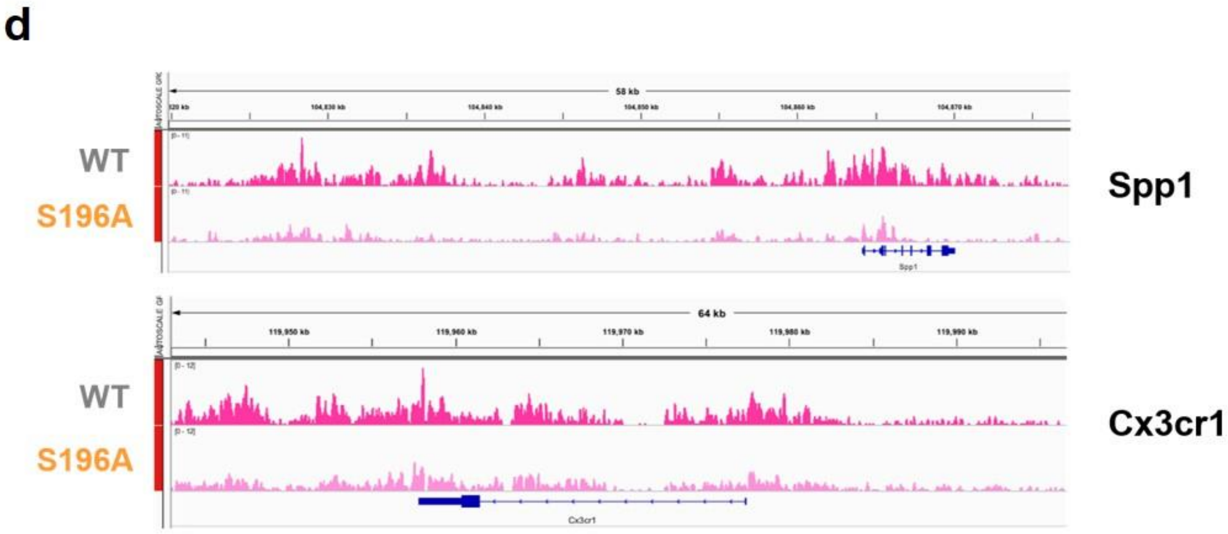

(a) Total spectral counts obtained from immunoprecipitates of wild-type human LXRα (LXRα), phospho-mutant (S198A) and control cells (expressing only the empty retroviral vector, VO) identified by mass spectroscopy. **(b)** Immunoprecipitation assays with cells expressing FLAG-tagged wild-type human LXRα (LXRα) and S198A (SA) mutant or vector only (VO). Wild-type and mutant LXRα were immunoprecipitated with anti-FLAG agarose beads followed by immunoblotting with specific TBLR1 antibodies. Expression of TBLR1 and LXRα in protein extracts prior to immunoprecipitation analysis (input) are shown. **(c)** Hepatic triglycerides (TGs) from WT and S196A mice treated with vehicle (Veh) or 50 mg/kg T0901317 (T1317). Values shown normalised to protein levels. **(d)** Representative H3K27Ac ChIP-seq read alignment tracks in WT and S196A HFHC-fed livers for genes shown to be reduced in S196A mice.

Table S1. Related to Figure 1.

Biometric and metabolic parameters of mice fed a chow diet.

Parameter	Genotype	Mean ± SEM	*p*
**Body weight (grams)**	WT<br>S196A	23.63 ± 0.6<br>21.70 ± 0.75	0.132
**% Liver weight (Liver g/Body g)**	WT<br>S196A	4.69 ± 0.25<br>4.41 ± 0.07	0.241
**Plasma glucose (mmol/L)**	WT<br>S196A	5.35 ± 0.10<br>4.63 ± 0.22	0.268
**Plasma insulin (ng/ mL)**	WT<br>S196A	0.34 ± 0.05<br>0.87 ± 0.24	0.103
**Hepatic triglycerides (µg / mg protein)**	WT<br>S196A	51.95 ± 5.06<br>37.63 ± 4.50	0.116
**Hepatic total cholesterol (µg / mg protein)**	WT<br>S196A	98.96 ± 10.48<br>104.43 ± 4 .05	0.688

Table S2. Related to Figure 1.

Biometric and metabolic parameters of mice fed a high fat and high cholesterol diet.

Parameter	Genotype	Mean ± SEM	p-value
**Body weight (grams)**	**WT**<br>**S196A**	21.36 ± 0 .41<br>19.89 ± 0.35	**0.012**
**% Liver weight (Liver g/Body g)**	**WT**<br>**S196A**	9.30 ± 0.17<br>6.41 ± 0.18	**3.06E-12**
**Plasma glucose (mmol/L)**	**WT**<br>**S196A**	4.49 ± 0.30<br>4.61 ± 0.24	0.762
**Plasma insulin (ng/ mL)**	**WT**<br>**S196A**	0.60 ± 0.10<br>0.87 ± 0.33	0.498
**Hepatic triglycerides (µg/ mg protein)**	**WT**<br>**S196A**	106.66 ± 10.04<br>170.41 ± 23.85	**0.033**
**Hepatic total cholesterol (µg/ mg protein)**	**WT**<br>**S196A**	207.79 ± 23.28<br>53.33 ± 2.76	**0.00006**

Table S3. Related to STAR Methods.

	**Forward primer** (5' to 3')	**Reverse primer** (5' to 3')
Adipophilin	GACCGTGCGGACTTGCTC	GCCATTTTTTCCTCCTGGAGA
Abca1	GGACATGCACAAGGTCCTGA	CAGAAAATCCTGGAGCTTCAAA
Abcg1	CCTTCCTCAGCATCATGCG	CCGATCCCAATGTGCGA
Abcg5	TGGATCCAACACCTCTATGCTAAA	GGCAGGTTTTCTCGATGAACTG
Abcg8	TGCCCACCTTCCACATGTC	ATGAAGCCGGCAGTAAGGTAGA
a-Sma	CCCAGACATCAGGGAGTAATGG	TCTATCGGATACTTCAGCGTCA
Atf3	GAGGATTTTGCTAACCTGACACC	TTGACGGTAACTGACTCCAGC
Atp6v0d2	GTGCAGTGTGAGACCTTGGA	GCCAGGAAGTTGCCATAGTC
Bex1	ATGGAGTCCAAAGATCAAGGCG	CTGGCTCCCTTCTGATGGTA
Cd36	GCCAAGCTATTGCGACATGA	TCTCAATGTCCGAGACTTTTCAAC
Ces1f	TGGAGAGTCAGCAGGAGGTT	ATGAAGGCCACACCACTCTC
Chop	CTGGAAGCCTGGTATGAGGAT	CAGGGTCAAGAGTAGTGAAGGT
Col1a1	GCTCCTCTTAGGGGCCACT	CCACGTCTCACCATTGGGG
Cyclophylin	GGCCGATGACGAGCCC	TGTCTTTGGAACTTTGTCTGCAA
Cyp17a1	ACCAGCCAGATCGGTTTATG	AGGGCAAATAACTGGGTGTG
Cyp2b13	ATGCTCATGTACCCCCATGT	GCCGATCACCTGATCAATCT
Cyp2b9	CTGGCCACCATGAAAGAGTT	CATTGGGCCTCCTCCTTTAT
Cyp2c69	CACAGTGGCTCATGAAGGAA	GATGAATTGGGGATCACAGG
Cyp7a1	CATACCTGGGCTGTGCTCTG	CCAAATGCC TTCGCAGAAGATG
Cx3cr1	TGAGTGACTGGCACTTCCTG	AAGGAGGTGGACATGGTGAG
Dgat2	CTGGCTGATAGCTGCTCTCTACTT	TGTGATCTCCTGCCACCTTTC
Elovl6	TGAACAAGCGAGCCAAGTTTG	GAGCACCGAATATACTGAAGACG
Fabp5	AGGATGGGAAGATGATCGTG	CTGGCAGCTAACTCCTGTCC
Fas	GCTGCGGAAACTTCAGGAAAT	AGAGACGTGTCACTCCTGGACTT
Fsp27	GTGTCCACTTGTGCCGTCTT	CTCGCTTGGTTGTCTTGATT
Hamp2	AGAAAGCAGGGCAGACATTG	GCAGATGGGGAAGTTGATGT
Idol	ATGCTGTGCTATGTCACGAGG	TCGATGATCCCTAGACGCCTG
Ldlr	GCATCAGCTTGGACAAGGTGT	GGGAACAGCCACCATTGTTG
L-Fabp	ATGAACTTCTCCGGCAAGTACC	CTGACACCCCCTTGATGTCC
Lpcat3	CCTTCACGGGCCTCTCAATT	CCATGAGTCGCAGGATGAGG
Lxra	GGTTGCTTTAGGGATAGGGTT	TTCCGCTTTTGTGGACGAAG
Nnmt	TGTGCAGAAAACGAGATCCTC	TGTGCAGAAAACGAGATCCTC
Ncp1l1	GAGAGCCAAAGATGCTACTATCTT	CCCGGGAAGTTGGTCATG
Osbpl3	AGACACGGAGGAGCACATCT	CGGTACATTCTGTGGTGACG
Osm	GCAGCTGTGGCTTTCTCTGG	TCGTCCCATTCCCTGAAGAC
Ppp1r3g	CTGAGACCCCGATCCCTGAT	GAGAGCGGCGATATTCCTGT
Scara3	GCTGGTGAAGACGAGGACAT	CAAAATCCGCACTGATGTGT
Scd1	CCGGAGACCCCTTAGATCGA	TAGCCTGTAAAAGATTTCTGCAAA
Slc22a26	ACAGAGCCCTGTATGGATGG	AGATCCACACACCAGGTTCC
Srebp1c	CAGGAGGACATC TTGCTGCTTC	TTGGGAGGCTGGTTTTGACC
Syngr1	CTGGTTCGTGGGTTTCTGCTT	GTCCCTTCGTTCAGAGGGTTG
Tgfb2	TTTGCTCCAGACAGTCCCAG	ATCTCCAGACATGCCAAGCC
Timp1	GTGGATATGCCCACAAGTCC	CTCAGAGTACGCCAGGGAAC
Thrsp	GCGGAAATACCAGGAAATGA	CGGGGTCTTCATCAGTCTTC
spl Xbp1	GAGTCCGCAGCAGGTG	GTGTCAGAGTCCATGGGA
Stk25	ACGTT CCTCCAACCATCCG	CTTCTGTGAGCTGTGCA
Wfdc3	CTTGGGTAGCTGCAGGAGAG	ATTCGTCTCCGGTACACAGC

Table S4. Related to STAR Methods.

	**Forward primer** (5' to 3')	**Reverse primer** (5' to 3')
Ces1f DR4	GGTGGTGGCCATTCAATATC	TGTCCACAAACCCTACCTGA
Ces1f TSS	CATTGACTTGGGAGCCTGTC	ACTCACCGCAAATCACACAG
Srebp1c LXRE	AGGCTCTTTTCGGGGATGG	TGGGGTTACTGGCGGTCAC
Srebp1c TSS	GTGGGCCTAGTCCGAAGC	ATCTCGGCCAGTGTCTGTTC
